# Comparative transcriptome investigation of global gene expression changes caused by miR156 overexpression in *Medicago sativa*

**DOI:** 10.1186/s12864-016-3014-6

**Published:** 2016-08-19

**Authors:** Ruimin Gao, Ryan S. Austin, Lisa Amyot, Abdelali Hannoufa

**Affiliations:** 1 Agriculture and Agri-Food Canada, 1391 Sandford Street, London, ON N5V 4T3 Canada; 2 Department of Biology, University of Western Ontario, 151 Richmond Street, London, ON N6A 5B7 Canada

**Keywords:** *Medicago sativa*, RNA-Seq, Transcriptome, miRNA, *SPL*, Biomass

## Abstract

**Background:**

*Medicago sativa* (alfalfa) is a low-input forage and potential bioenergy crop, and improving its yield and quality has always been a focus of the alfalfa breeding industry. Transgenic alfalfa plants overexpressing a precursor of alfalfa *microRNA156* (*MsmiR156*) were recently generated by our group. These plants (miR156OE) showed enhanced biomass yield, reduced internodal length, increased shoot branching and trichome density, and a delay in flowering time. Transcripts of three *SQUAMOSA-PROMOTER BINDING PROTEIN-LIKE (SPL)* genes (*MsSPL6*, *MsSPL12*, and *MsSPL13*) were found to be targeted for cleavage by *MsmiR156* in alfalfa.

**Results:**

To further illustrate the molecular mechanisms underlying the effects of miR156 in alfalfa, two miR156OE genotypes (A11a and A17) were subjected to Next Generation RNA Sequencing with Illumina HiSeq. More than 1.11 billion clean reads were obtained from our available sequenced samples. A total of 160,472 transcripts were generated using Trinity *de novo* assembly and 4,985 significantly differentially expressed genes were detected in miR156OE plants A11a and A17 using the *Medicago truncatula* genome as reference. A total of 17 genes (including upregulated, downregulated, and unchanged) were selected for quantitative real-time PCR (qRT-PCR) validation, which showed that gene expression levels were largely consistent between qRT-PCR and RNA-Seq data. In addition to the established *SPL* genes *MsSPL6*, *MsSPL12* and *MsSPL13*, four new *SPLs; MsSPL2*, *MsSPL3*, *MsSPL4* and *MsSPL9* were also down-regulated significantly in both miR156OE plants. These seven *SPL* genes belong to genes phylogeny clades VI, IV, VIII, V and VII, which have been reported to be targeted by *miR156* in *Arabidopsis thaliana*. The gene ontology terms characterized electron transporter, starch synthase activity, sucrose transport, sucrose-phosphate synthase activity, chitin binding, sexual reproduction, flavonoid biosynthesis and lignin catabolism correlate well to the phenotypes of miR156OE alfalfa plants.

**Conclusions:**

This is the first report of changes in global gene expression in response to miR156 overexpression in alfalfa. The discovered miR156-targeted *SPL* genes belonging to different clades indicate miR156 plays fundamental and multifunctional roles in regulating alfalfa plant development.

**Electronic supplementary material:**

The online version of this article (doi:10.1186/s12864-016-3014-6) contains supplementary material, which is available to authorized users.

## Background

*Medicago sativa* (alfalfa) is a perennial forage legume that is also a candidate low-input bioenergy crop due to its great yield potential and high energy value [1–3]. However, in order to fully realize alfalfa’s potential, significant improvements to biomass yield and quality are needed to compete against high yielding grasses, such as switchgrass and miscanthus. Recently, we overexpressed a precursor of *miRNA156* (*MsmiR156*) in alfalfa, and this led to up to a 2-fold increase in biomass yield, delayed flowering time, enhanced cellulose content and reduced lignin, producing an overall improvement in biomass quality [4]. In addition, three *SQUAMOSA-PROMOTER BINDING PROTEIN-LIKE (SPL)* genes (*MsSPL*6, *MsSPL12*, and *MsSPL13*) were found to be downregulated via transcript cleavage by miR156 in alfalfa [4]. All SPL proteins constitute a family of transcription factors which contain a highly conserved DNA binding domain of 76 amino acids (called SBP domain) with two zinc binding sites and a nuclear localization signal (NLS) [5]. Given the diversity of traits affected by overexpression of *miR156* in alfalfa, it is critical to identify and characterize its downstream target genes, especially *SPL* genes and genes that are regulated by *SPLs*, as well as understand the functions and behaviours of *SPL* genes and their target genes by solidly linking each to one or more phenotypes exhibited by miR156OE plants.

MiR156 and its *SPL* target genes play crucial roles in regulating different aspects of plant growth and development [6–10]. Although some similarities are shared among the same clade of *SPL* genes, many of the *SPL* genes from the same clade possess different functions in different plant species. For example, *AtSPL2*, *AtSPL10,* and *AtSPL11* are involved in controlling leaf shape, regulating shoot maturation and stimulating trichome production in *Arabidopsis thaliana* [11]. In addition, repression of *AtSPL2* and *AtSPL11* by *miR156* is also required for heat stress memory [12]. *AtSPL3*, *AtSPL4* and *AtSPL5* are involved in prolonging developmental transition and delaying flowering time [13]. *AtSPL9* and *AtSPL15* mainly promote shoot maturation, delayed flowering, increased anthocyanin accumulation, and sensitivity to stress treatment, as well as enhanced carotenoid accumulation in the seed [14–17]. In rice (*Oryza sativa*), *OsSPL3, OsSPL4, OsSPL11, OsSPL13* and *OsSPL14* can increase anthocyanin accumulation and tiller number, and promote panicle branching and grain yield [16, 18, 19]. *OsSPL16* controls rice grain size, shape and quality [20]. Furthermore, a genomic organization study found that *OsSPL2, OsSPL3, OsSPL4, OsSPL7, OsSPL11, OsSPL12, OsSPL13, OsSPL14, OsSPL16, OsSPL17* and *OsSPL18* increase tiller numbers, delay flowering, reduce the number of spikelets and grains per panicle, as well as decrease secondary branches of panicles [21]. In maize, the *SPL* homologue *TEOSINTE GLUME ARCHITECTURE1* can prolong developmental phase transition and delay flowering [22]. In potato (*Solanum tuberosum*), *StSPL3*, *StSPL6*, *StSPL9*, *StSPL13* and *LIGULELESS1* affect plant architecture and tuberization [23]. In *Lotus japonicus*, miR156-targeted genes, *SPLs* and *WD40,* can prolong developmental phase transition, delay flowering time and enhance shoot branching [24]. In switchgrass (*Panicum virgatum* L.), *PvSPL1, PvSPL2, PvSPL3* and *PvSPL6* enhance shoot branching and increase biomass production and forage quality [25].

In recent years, genome-wide global transcriptome analysis has become a powerful tool to uncover genes which control various traits in plants. For example, using transcriptome analysis, Zhou et al. (2014) discovered a candidate MYB transcription factor responsible for red leaf coloration in peaches [26]. From the *de novo* assembled *Ipomoea nil* (morning glory) transcriptome, genes in the phenylpropanoid biosynthesis pathway were identified and SSR markers were developed for deployment in breeding programs [27]. Transcriptome analysis of *Lotus corniculatus* identified genes involved in secondary metabolism [28]. Comparative transcriptome analysis of latex from two different rubber tree clones (*Hevea brasiliensis* Muell. Arg.) revealed new cues for the regulation of latex regeneration and duration of latex flow [29]. Similarly, in alfalfa, transcriptome analysis of resistant and susceptible alfalfa cultivars infected with root-knot nematode unveiled a number of differentially expressed common and cultivar-specific genes [30]. Identification of candidate genes related to fall dormancy in dormant and non-dormant alfalfa cultivars was also accomplished by analyzing the leaf transcriptomes of these two cultivars [31]. The *M. sativa* gene index 1.2 was used to investigate gene expression differences between *M. sativa* ssp. *sativa* (B47) and *M. sativa* ssp. *falcata* (F56) [32]. So far, there has been no reported transcriptome analysis for miR156OE alfalfa plants; however, using microarray hybridization, Xie et al. reported that the expression levels of 3008 genes were affected in leaves of miR156OE rice (*Oryza sativa*) [33]. Thus, analysis of genome-wide changes in gene expression profiles in contrasting alfalfa cultivars should not only reveal differentially expressed genes, but also provide insights into possible molecular mechanisms that underlie various traits in miR156OE alfalfa plants. These include delayed flowering time, enhanced biomass production, and increased shoot branching [4].

To illustrate changes in global gene expression induced by miR156 overexpression in alfalfa, we conducted RNA sequencing (RNA-Seq) on two miR156OE alfalfa genotypes (A11a and A17) generated in our previous study, and which showed reduced plant height and stem thickness; increased branching (main and lateral branches) and node numbers, as well as increased trichome density in leaves [4]. In addition, these plants showed delayed flowering time, a reduction in lignin content and an increase in cellulose content compared to WT control [4]. The present analysis investigates whether additional *SPL* genes are targeted for transcript cleavage by miR156, and what other genes are differentially expressed in miR156OE alfalfa plants.

## Results

### A d*e novo* assembled alfalfa transcriptome

**Fig. 1:**
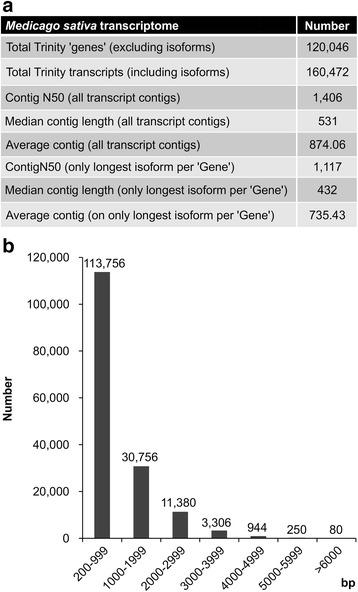
Statistics summary of *de novo* assembly of *Medicago sativa* transcriptome using the Trinity program. **a** Summary of *de novo* assembly of *M. sativa* transcriptome. **b** Length distribution of *de novo* assembled transcripts

In order to illustrate the role of miR156 in alfalfa plant development, WT and the two most prominent miR156OE genotypes A11a and A17 [4] were selected for Next Generation Sequencing at the transcriptome level to detect differentially expressed genes (DEGs). Since the full sequence of the alfalfa genome has not been reported yet, the *de novo* assembled alfalfa transcriptome was first obtained using all available HiSeq data sequenced by our group. After filtering for low-quality and problematic reads such as empty adapters, short reads and unpaired reads, 1.11 billion reads were assembled using the Trinity transcriptome assembly program [34]. A total of 160,472 transcripts ranging in size from 200 bp to 9673 bp were obtained (Additional file 1: Table S1) with an average length of 874.06 bp and an N50 of 1406 bp (Fig. 1). These represented 120,046 Trinity ‘genes’ with an average length of 735.43 bp and an N50 of 1117 bp (Fig. 1a). The majority (113,756) of these genes were in the range of 200–999 bp (Fig. 1b).

### Differentially expressed genes in miR156OE genotypes

**Fig. 2:**
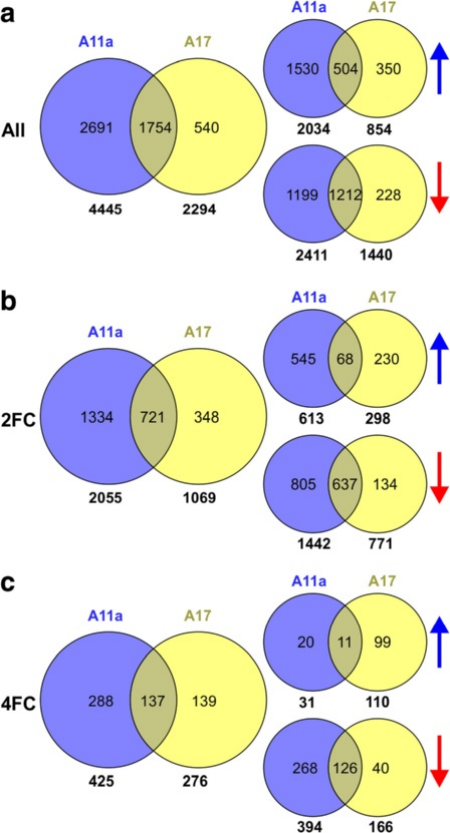
Numbers of differentially expressed genes (DEGs) from RNA-Seq data between *M. sativa* WT control and miR156OE genotypes, A11a and A17, using Tophat-cufflinks analysis based on the *M. truncatula* genome. Total number of genes detected with (**a**) significant (**b**) two-fold change (FC) and (**c**) four-FC from leaves of WT control and miR156OE A11a and A17 in *M. sativa.* The overlapping gene numbers between the three different categories are shown in the Venn diagrams. The *blue* and *red arrows* represent upregulation and downregulation, respectively

As miR156 overexpression affects a number of traits in alfalfa plants [4], we set out to identify DEGs that may be responsible for such traits. In total, relative to WT, 4,445 genes were significantly affected in genotype A11a which was roughly twice that observed in genotype A17 which had 2,294 DEGs (*p* < 0.005) (Additional file 2: Table S2). Of all DEGs, 1,754 (502 up-regulated and 1,212 down-regulated) were differentially expressed in both genotypes (Fig. 2a). In order to narrow down the gene list, we focused on DEGs with at least a 2-fold change (FC). There were 2,055 (613 up-regulated and 1,442 down-regulated) and 1,069 (298 up-regulated and 771 down-regulated) DEGs with a 2 FC for A11a and A17, respectively, of which 721 (68 up-regulated and 637 down-regulated) overlapped between the two genotypes (Fig. 2b). Moreover, there were 425 (31 up-regulated and 394 down-regulated) and 276 (110 up-regulated and 166 down-regulated) DEGs with at least a 4 FC in genotypes A11a and A17, respectively, of which 137 (11 up-regulated and 126 down-regulated) overlap between the two genotypes (Fig. 2c).

### Gene ontology (GO) enrichment analysis of DEGs

**Fig. 3:**
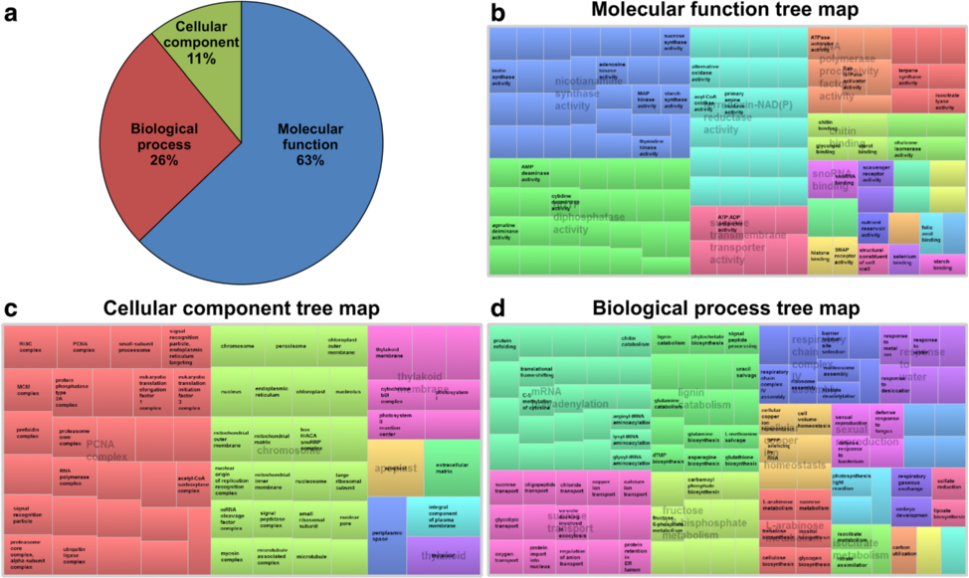
Gene Ontology (GO) enrichment analysis of DEGs related to *M. sativa* development. (**a**) Fractional distribution of differentially expressed GO terms based on molecular function, cellular component and biological process. Tree maps of (**b**) molecular function, (**c**) cellular component and (**d**) biological process of 2 FC DEG GO terms

Gene ontology enrichment analysis was carried out to identify pathways that may be affected in miR156OE plants. Combining all of the >2FC DEGs from genotypes A11a and A17, approximately 12,514 GO terms were assigned (Fig. 3a). Many of these GO terms could reflect some traits affected by *miR156* overexpression. Of the 132 GO terms in the molecular function category; nicotianamine synthase activity, ferredoxin-NAD(P) reductase activity, transcription regulatory region sequence-specific DNA binding, folic acid binding, starch synthase activity, starch binding, sucrose-phosphate synthase activity, chitin binding and electron transporter (transferring electrons within the cyclic electron transport pathway of photosynthesis activity) (Fig. 3b) are of particular interest. For example, many transcription factors (such as *SPL* genes) which can bind specific DNA sequences [35] were found to be significantly down-regulated, and this is closely related to the term of transcription regulatory region sequence-specific DNA binding. The GO terms cellular component, thylakoid, thylakoid membrane and chromosome (Fig. 3c) may be related to photosynthesis, which could affect miR156OE traits, such as elevated biomass production, and influence biosynthesis of sugar, starch and lignin [4]. Among the 17 functions classified as biological processes; response to water, sexual reproduction, flavonoid biosynthesis, sucrose transport, cellular copper ion homeostasis, and lignin catabolism (Fig. 3d) are the main interesting terms because they are related to the miR156OE traits such as delayed flowering time and effects on sugar, starch, lignin and cellulose contents [4, 9]. The full list of the components for the three fractions (molecular function, cellular component and biological process) is shown in Additional file 3: Table S3.

### Validation of RNA-Seq data by quantitative real time PCR

To validate the RNA-Seq data, we randomly selected 17 genes (including upregulated, downregulated, and unchanged) for expression analysis by quantitative real time PCR (qRT-PCR). All of the qRT-PCR primers were designed based on alfalfa transcripts which were assembled using the Trinity program (Additional file 1: Table S1). The expression levels of the selected genes from the RNA-Seq analysis were compared to the qRT-PCR data in Table 1. In general, there was a strong correlation between the two sets of expression data. All genes selected for validation showed a similar expression trend (up-regulation, down-regulation, or unchanged) in the qRT-PCR and RNA-Seq analysis, and 14 of the 17 transcripts (82 %) displayed a similar level of expression change (Table 1). These results support a strong level of confidence in our RNA-Seq data.

**Table 1:** Validation of RNA-Seq data using qRT-PCR

Gene name	Samples	DEG (FC)	qRT-PCR (FC)
MADS-box transcription factor	WT/A17	−48.5947	−43.5837
(E)-beta-ocimene/myrcene synthase	WT/A11a	−11.8553	−31.4647
Floral meristem identity control protein LEAFY (LFY) protein	WT/A11a	−18.1674	−20.9683
Double-stranded RNA-binding motif protein	WT/A11a	−5.2929	−15.9901
Squamosa promoter-binding-like protein 9	WT/A11a	−2.5015	−3.9542
Thioredoxin-like protein	WT/A11a	−52.4798	−2.5392
Sesquiterpene synthase	WT/A11a	−45.1872	−4.7343
DOF-type zinc finger DNA-binding family protein	WT/A11a	−14.9880	−4.3208
Filament-like plant protein	WT/A17	0.9325	0.9876
RecA-like protein	WT/A11a	1.2135	1.0700
Histidine kinase-, DNA gyrase B, putative	WT/A17	1.0408	0.9876
Rho-like GTP-binding protein	WT/A17	1.0373	0.9875
Coiled-coil protein	WT/A11a	1.0222	0.9875
Ribonuclease T2 family protein	WT/A17	13.8756	32.8815
NAC transcription factor-like protein	WT/A17	34.9484	39.4085
Homeobox leucine zipper protein	WT/A11a	26.1393	39.4371
Uridylate kinase-like protein	WT/A11a	23.6090	1.8065

### Novel *SPL* targets of miR156 in alfalfa

**Fig. 4:**
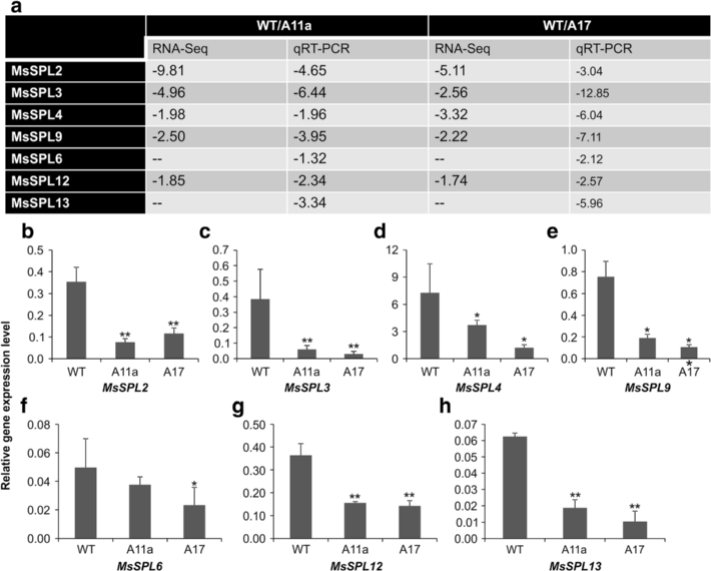
Validation of the *miR156* targeted *SPL* genes in miR156OE plants using qRT-PCR. (**a**) Fold change comparison of transcript levels of seven *SPL* genes between NGS results and qRT-PCR validation, “--” indicates the FC was not significant to be detected. The relative transcript level of the newly discovered *SPLs* (**b**) *MsSPL2*, (**c**) *MsSPL3*, (**d**) *MsSPL4*, and (**e**) *MsSPL9*. The relative transcript level of the previously established *SPLs* (**f**) *MsSPL6*, (**g**) *MsSPL12*, and (**h**) *MsSPL13*. Relative gene transcript levels were analyzed using the 2^-∆CT^ method. Means of three independent biological repeats were used in this study. The student *t* test was used to analyze the significant differences of each tested gene between WT and miR156OE genotypes A11 and A17 (*p < 0.05, **p < 0.01)

In addition to the three previously reported miR156-targeted *SPL* genes in alfalfa [4], our RNA-Seq analysis revealed four more significantly down-regulated *SPL* genes in both miR156OE genotypes, namely *MsSPL2*, *MsSPL3*, *MsSPL4*, and *MsSPL9* (Additional file 2: Table S2), which are homologous to *M. truncatula SPL* genes Medtr8g463140, Medtr2g014200, Medtr4g088555 and Medtr7g092930, respectively*.* We further tested the expression patterns of the four *SPLs* by qRT-PCR and found that the transcript fold changes were consistent with those detected by RNA-Seq (Figs. 4a, b, c, d and e). Similarly, RNA-Seq and qRT-PCR data showed that the previously reported *MsSPL12* was also down-regulated in the miR156OE genotypes A11a and A17 (Fig. 4a and g). Conversely, *MsSPL6* and *MsSPL13* were not detected with significant downregulation in the RNA-Seq analysis (Fig. 4a); however, their significant down-regulation in A11a and A17 was detected using qRT-PCR (Fig. 4f and h). In summary, a total of seven *SPL* genes are significantly down-regulated in miR156OE genotypes. These genes are thus potential targets for transcript cleavage by miR156 in alfalfa.

**Fig. 5:**
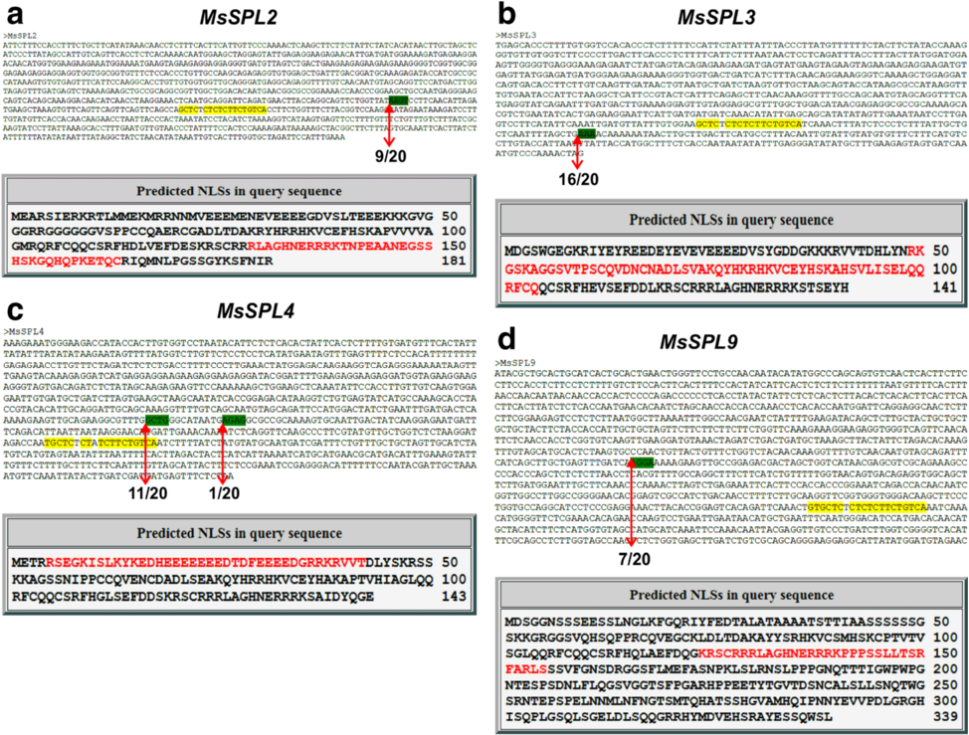
Validation of the *miR156* cleavage sites in *MsSPL2/3/4* and *MsSPL9* transcripts and prediction of the Nuclear Localization Signal. The predicted *miR156* target sequence (*highlighted in yellow*) was located in the 3’ untranslated region of (**a**) *MsSPL2,* (**b**) *MsSPL3,* and (**c**) *MsSPL4* and (**d**) the open reading frame region of *MsSPL9*. Cleavage sites are highlighted in *green*. Denominators refer to the number of clones sequenced whereas the nominators represent the number of clones cleaved at a particular site. The NLS prediction was carried out based on http://nls-mapper.iab.keio.ac.jp/cgi-bin/NLS_Mapper_form.cgi and the corresponding nucleotide sequence is indicated in *red text*

To investigate whether *miR156* directly targets the four newly discovered *SPL* genes, we identified their predicted *miR156* recognition sites using sequence alignment and used a modified 5’-RACE technique [4] to test for transcript cleavage. Among the twenty clones sequenced for each gene, transcript cleavage was detected in all four *SPLs* outside of their predicted miR156 target sites: 54 bp upstream in nine *MsSPL2* clones (Fig. 5a), 42 bp downstream in sixteen *MsSPL3* clones (Fig. 5b), 143 bp upstream in eleven clones and 130 bp upstream in one clone of *MsSPL4* (Fig. 5c), and 350 bp upstream in seven *MsSPL9* clones (Fig. 5d). We also found that each of the four *SPLs* has a predicted nuclear localization signal according to the online software prediction (http://nls-mapper.iab.keio.ac.jp/cgi-bin/NLS_Mapper_form.cgi) (Fig. 5).

### Phylogeny of *MsSPL* genes in alfalfa

**Fig. 6:**
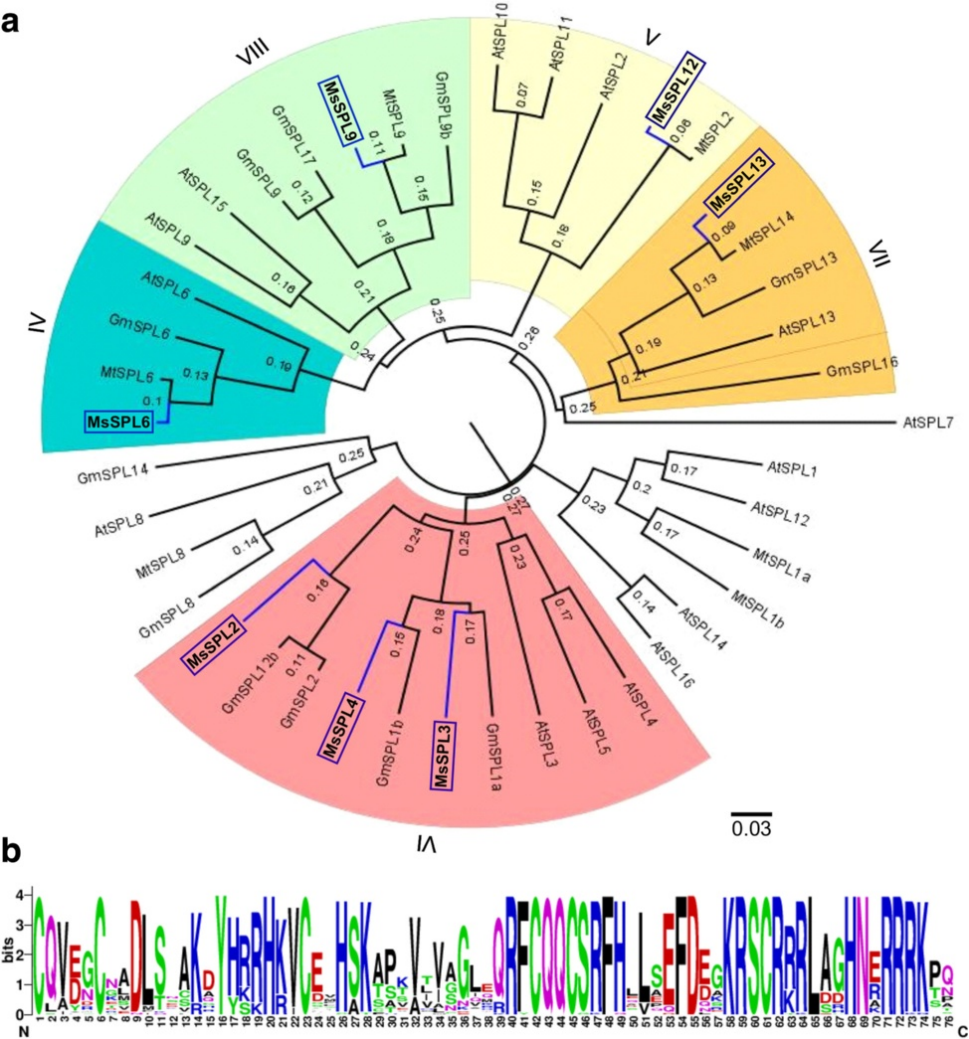
Phylogenetic analysis of differentially expressed *SPL* genes in miR156OE *M. sativa* plants. **a** Phylogenetic tree based on an alignment of the SBP-box domain in *SPLs* from *M. sativa*, *M. truncatula*, Arabidopsis*,* and *Glycine max. M. sativa SPLs (MsSPLs)* are highlighted with *blue box*. **b** A representation of the conserved amino acids in the SBP domain from the genes included in the phylogenetic tree. The height of the letter (amino acid) at each position represents the degree of conservation

The conserved SBP domain was used to generate a phylogenetic tree for the *SPL* gene family in *M. sativa* and its close relatives *M. truncatula* and *Glycine max*, as well as the model plant Arabidopsis*.* The *SPL* genes can be grouped into eight main clades (Fig. 6a). Clades I, II and III represent *SPL* genes that are not targeted by *miR156* (not highlighted with colour in Fig. 6a), while genes from clades IV, V, VI, VII and VIII can undergo cleavage by *miR156.* The multitude of traits affected in alfalfa by *miR156* overexpression could be explained by the fact that its *SPL* targets, i.e. *MsSPL2/3/4*, *MsSPL6*, *MsSPL9*, *MsSPL12* and *MsSPL13,* belong to clades VI, IV, VIII, V and VII, respectively (Fig. 6a). *In silico* analysis of the SBP domains that were used to generate our phylogenetic tree showed that six cysteines, four histidines, and eight arginines were absolutely conserved (Fig. 6b). The nucleotide sequence of the SBP domain is also shown in Additional file 4: Figure S1.

### *MsmiR172* is downregulated in miR156OE alfalfa plants

*MiR156* and *miR172* signals are integrated at the *SPL3, SPL4, SPL5* and *SPL9* genes in the model plant Arabidopsis [36, 37]. Consistent with this finding, our RNA-Seq data revealed that the *M. sativa miR172* precursor, *MsmiR172* (which is homologous to *M. truncatula* Medtr2g101400), was significantly downregulated in both miR156OE genotypes (*p* < 0.00005) (the downregulation FC for genotype A11a and A17 are 11.09 and 8.33, respectively) relative to the WT control (Additional file 2: Table S2). In addition, three *miR172-*targeted genes homologous to Medtr7g117690, Medtr7g100590 and Medtr2g093060, which encode AP2 domain transcription factors [38], were significantly downregulated in both A11a and A17 genotypes (Additional file 2: Table S2). Whereas the *MsmiR172* precursor was shown to be 76.82 % identical to its *M. truncatula* homologue, the mature sequence was completely conserved (Additional file 5: Figure S2).

### Tissue-specific expression of *miR156*, *miR172,* and miR156-targeted *SPL* genes in alfalfa

**Fig. 7:**
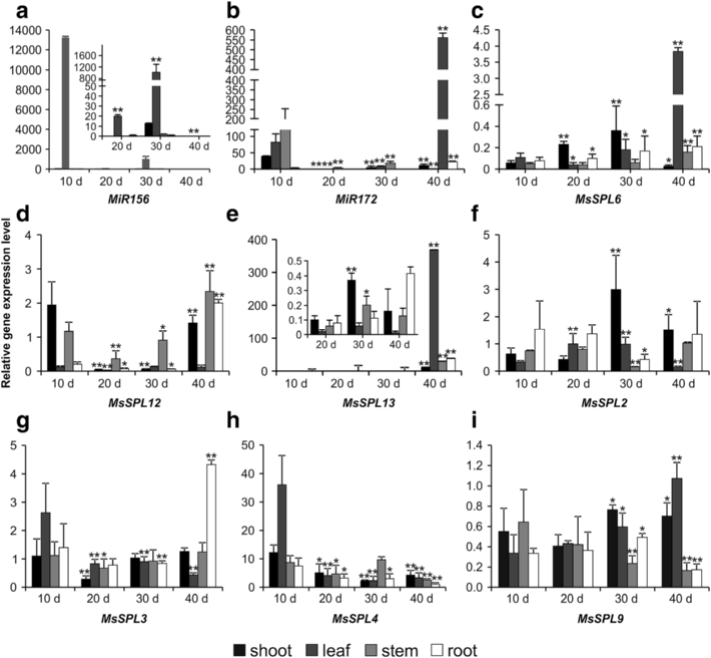
Developmental and tissue-specific expression profiles of *miR156, miR172*, and *miR156*-targeted *SPL* genes in *M. sativa*. Relative gene transcript levels of (**a**) *miR156,* (**b**) *miR172*, (**c**) *MsSPL6*, (**d**) *MsSPL12*, (**e**) *MsSPL13,* (**f**) *MsSPL2*, (**g**) *MsSPL3*, (**h**) *MsSPL4* and (**i**) *MsSPL9* were analyzed by the 2^-∆CT^ method. Means of three independent biological repeats were used to examine tissue-specific expression for each gene at 20, 30, and 40 days compared to the 10-day time point (*p < 0.05, **p < 0.01)

To gain an insight into how *miR156* and its target genes are regulated in alfalfa, we evaluated the expression of *miR156,* its target *SPL* genes, and *miR172* in four tissue types at different developmental time points from the juvenile stage (10 day-old rooted cuttings) to just before flowering. Expression analysis showed that *miR156* was primarily expressed in the leaves, with the highest levels observed at the earliest time point, 10 days (Fig. 7a). In contrast, *miR172* could be detected in all tissue types, except for roots, at 10 days, with the highest levels observed in stems, just before flowering, at 40 days (Fig. 7b). In general, *MsSPL6* and *MsSPL13* had an opposite expression pattern to *miR156,* with the highest transcript levels observed in the leaves at 40 days (Fig. 7c, e). On the other hand, *MsSPL12* (Fig. 7d)*, MsSPL2* (Fig. 7f)*, MsSPL3* (Fig. 7g)*, MsSPL4* (Fig. 7h) and *MsSPL9* (Fig. 7i) had diverse expression profiles*.* For example, *MsSPL3* was expressed most strongly in roots at 40 days.

### Altered expression of flowering pathway-related genes in miR156OE alfalfa

**Fig. 8:**
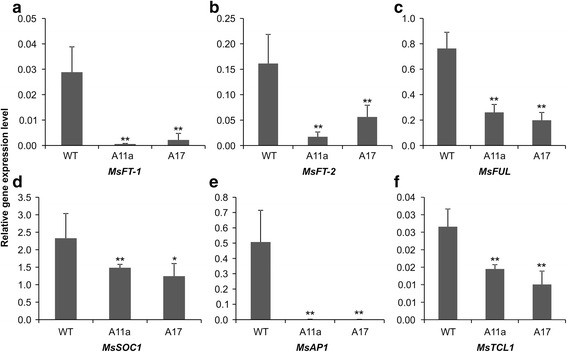
qRT-PCR validation of potential downstream SPL-target genes that affect flowering time in miR156OE alfalfa plants. Relative transcript levels of (**a**) *MsFT-1,* (**b**) *MsFT-2,* (**c**) *MsFUL,* (**d**) *MsSOC1* and (**e**) *MsAP1,* and (**f**) *MsTCL1.* Means of three independent biological repeats were used in this study. The Student’s *t* test was used to analyze the significant differences of each of the tested genes between WT and miR156OE genotypes A11a and A17 (*p < 0.05, **p < 0.01)

Our RNA-Seq data revealed that several flowering-related genes, including *LEAFY (LFY)*, *FLOWERING LOCUS T (FT)*, *FRUITFULL (FUL)*, *SUPPRESSOR OF OVEREXPRESSION OF CONSTANS1 (SOC1)* and *APETALA1 (AP1)*, were down-regulated in the leaves of miR156OE genotypes relative to the WT control (Additional file 2: Table S2, their corresponding gene IDs in *M. truncatula* are Medtr3g098560, Medtr7g006690, Medtr4g109830, Medtr7g075870 and Medtr8g066260). Transcript sequences of the aforementioned genes were obtained from our alfalfa *de novo* assembled transcriptome and then used to design qRT-PCR primers for gene expression analysis. All five genes were significantly downregulated in the miR156OE genotypes A11a and A17 relative to WT (Table 1 and Fig. 8a–f, two discovered homologous *MsFT-1* and *MsFT-2* in alfalfa). Since the above-mentioned flowering related genes are potentially regulated by *SPL* genes, the upstream 2000 bp of their promoter sequences were screened to examine if they contain the “GTAC” core sequence of SPL binding element, which can be specifically recognized by SBP domain [35]. Among these five genes, two of them, *LEY* and *FUL,* contain 5 and 6 “GTAC” elements, respectively (Additional file 6 Document 1).

## Discussion

We previously generated six alfalfa genotypes (A16, A8a, A8, A11, A17 and A11a) with increased miR156 expression [4]. Two of the genotypes, A17 and A11a (with a 1600- and 3400-fold increase in miR156, respectively), were chosen for RNA-Seq analysis. Compared to WT, these two genotypes displayed the most pronounced phenotypes, such as increased number of main/lateral branches and nodes, decreased plant height and internode length as well as delayed flowering, but the extent of these phenotypic changes was different between these two genotypes [4], presumably due to different miR156 levels. Our transcriptomic analysis, showed that overexpression of miR156 can affect some similar categories of downstream genes; genes differentially expressed in both genotypes, and which may affect similar phenotypes, but different miR156 expression levels could also affect expression of some unique genes in each genotype, which may affect the degree of phenotypic change relative to WT control. Therefore, it appears that diverse levels of miR156 expression may affect alfalfa traits differently.

Genes that are commonly downregulated in both A17 and A11a genotypes include four additional miR156-targeted *SPL* genes (*MsSPL2*, *MsSPL3*, *MsSPL4* and *MsSPL9*), in addition to the previously reported ones (*MsSPL6*, *MsSPL12* and *MsSPL13*) [4]. In Arabidopsis, *miR156* regulates 10 out of 16 Arabidopsis *SPL* genes that belong to the same clades as those silenced by miR156 in alfalfa [13, 39]. In addition, in both alfalfa and Arabidopsis, the *MsSPL2/3/4* and *AtSPL3/4/5* cleavage sites were found at the 3’UTR, and that of *MsSPL9* in the open reading frame region. However, unlike findings in Arabidopsis [13], cleavage sites for *MsSPL2/3/4* and *MsSPL9* in alfalfa were not detected within the predicted miR156 target region. In alfalfa, the detected cleavage sites were located either upstream or downstream of the predicted target sites. This may be explained by the RNA-induced silencing complex sliding through the transcript during miRNA-directed cleavage of the target [40]. Other studies have also shown similar results regarding cleavage site variation. For example, in a transcriptome-wide identification of miRNA targets using a degradome sequencing approach, a lower percentage of cleavage sites was found at the expected sequences for some conserved miRNAs in rice [41]. Also, *OsSPL14* was found to be cleaved by *OsmiR156* beyond its target site [18]. Although the cleavage sites for the newly discovered *MsSPL2*, *MsSPL3*, *MsSPL4* and *MsSPL9* in alfalfa are different from those in Arabidopsis, these *MsSPLs* belong to the same phylogenetic tree clades and share highly similar nucleotide and amino acid sequences with *AtSPLs* (Additional file 7: Figure S3 and Additional file 8: Figure S4), indicating these *SPLs* may perform similar functions in both alfalfa and Arabidopsis.

Based on the DEG list, the GO terms (such as effect of electron transporter, starch synthase activity, sucrose-phosphate synthase activity, chitin binding, sexual reproduction, flavonoid biosynthesis, sucrose transport and lignin catabolism) are closely related to miR156OE alfalfa phenotypes, namely enhanced shoot branching, delayed flowering time and elevated biomass production [4], which involve a large number of biological pathways. Among the differentially expressed *SPL* genes, we hypothesize that *MsSPL2/3/4* (clade VI) may perform similar functions as *AtSPL3/4/5* in Arabidopsis (Fig. 6a), because both of these two groups of *SPLs* are relatively small in size (420–550 bp) and contain complementary sequences of miR156 in the 3’ UTR. Leaves of Arabidopsis plants that overexpress *AtSPL3/4/5* can develop adult characteristics faster than WT control [13, 42]. *AtSPL3/4/5* also functions by integrating signals from the autonomous photoperiod, age and Gibberellic acid (GA) pathways to redundantly promote the reproductive transition [7, 43–48]. Under short day conditions, the three genes (*AtSPL3/4/5)* are negatively regulated by *miR156* in an age-dependent manner, and are positively regulated by *SOC1* through the GA pathway [36, 46]. Under long day conditions, however, *SOC1*, *FT*, and *FLOWERING LOCUS D (FD)* positively regulate *AtSPL3/4/5* in leaves in response to photoperiod signals [46]. SPL proteins are also known to indirectly activate *FT* expression through the direct binding of the inflorescence meristem gene *FUL*, and directly activate transcription of *FUL*, *AP1*, and *LFY* in the shoot apical meristem of Arabidopsis [44, 45, 49, 50]. Similarly, the above-mentioned flowering pathway-related genes (*LFY, FT, FUL, SOC1 and AP1*) were found to be significantly downregulated in miR156OE alfalfa plants (Fig. 8), suggesting that certain flowering mechanisms regulated by *SPLs* may be common in both Arabidopsis and alfalfa. In addition, some findings were also reported in snapdragon (*Antirrhinum majus*), where *AmSBP1*, an ortholog of *AtSPL3/4/5*, is involved in initiating flower development within the inflorescence [51]. Silencing of *AmSBP1* eliminates flowering completely and causes an increase in vegetative branching under long day conditions [51]. On the other hand, mutations in the *AtSPL3/4/5* ortholog *COLORLESS NON-RIPENING* resulted in fruits that failed to ripen [6], which may be considered as a novel function for *SPL* genes [5].

*MsSPL9*, discovered in this study, to be regulated by miR156 in alfalfa, belongs to clade VIII. This gene and its ortholog, *AtSPL15*, play redundant roles in regulating vegetative phase change and reproductive transition in Arabidopsis [14, 42]. Obvious phenotypes were observed in the double *spl9 spl15* Arabidopsis mutant with increased numbers of vegetative rosette leaves, rounder leaf shape and delayed flowering time compared to WT [14]. In addition, overexpression of *AtSPL9* in *hyponastic leaves1* mutants - which have lower *miR156* expression - caused complete loss of the juvenile phase [52]. Except for phase change, the plastochron length was also affected in the late flowering *atspl9spl5* double mutants, suggesting dissociation between growth and development [14]. Furthermore, genetic evidence indicates the involvement of *AtSPL9* in petal trichome initiation via activation of *TRICHOMELESS 1 (TCL1)* and anthocyanin pigment accumulation in vegetative stems [15, 53]. This *TCL1* gene was also significantly downregulated in our miR156OE alfalfa plants (Fig. 8f). In Arabidopsis and Patchouli (*Pogostemon acblin*), *SPL9* is involved in the regulation of sesquiterpene biosynthesis [54]. Furthermore, our qRT-PCR results show that the *SESQUITERPENE SYNTHASE* gene was downregulated in miR156OE plants (Table 1). *AtSPL9* and *AtSPL15* are two homologue genes in Arabidopsis and their functions are redundant, however, only one orthologue of *MsSPL9* was discovered in alfalfa (Fig. 6a). If this is the case, without the redundant homologous gene of *MsSPL15*, there will be an obvious phenotype in *MsSPL9* loss-of-function alfalfa genotypes. An ongoing project in our group is producing MsSPL9 overexpression as well as *MsSPL9* loss-of-function alfalfa genotypes. By investigating these transgenic alfalfa genotypes, we will be able to determine if *MsSPL9* also plays similar roles in regulating alfalfa vegetative phase change and reproductive transition, plastochron length, trichome development, anthocyanin pigment accumulation and sesquiterpene synthesis in alfalfa.

Expression analysis revealed that the transcripts of most of the *SPL* genes tested were detectable in roots. A recent publication reported that *AtSPL3*, *AtSPL9* and *AtSPL10* were involved in the repression of lateral root growth, and that miR156/SPLs module participates in lateral root primordia progression [55]. This is consistent with our results, which showed relatively high *SPL* transcript levels were detected in roots where miR156 transcript was undetectable.

## Conclusion

In summary, this is the first report on the effect of miR156 overexpression on global gene expression in alfalfa. At least 2403 genes were differentially expressed by at least 2-fold in miR156OE compared to WT alfalfa. Gene ontology analysis showed that the types of genes that are significantly regulated in miR156OE genotypes are closely related to biological processes that can impact the phenotypes observed in miR156OE alfalfa, including enhanced shoot branching, increased trichome density, a delay in flowering time and elevated biomass production. The *de novo* assembled alfalfa transcriptome will add to the limited publicly available alfalfa genomics resources, and will allow for easier identification of alfalfa gene sequences. Four additional *SPLs* (*MsSPL2/3/4* and *MsSPL9*) were discovered to be targeted for silencing by miR156 in alfalfa. Based on the phylogenetic tree analysis, all the current detected *SPL* genes in the miR156OE plants belong to five different clades, indicating that *miR156* plays fundamental and multifunctional roles in regulating alfalfa plant development. It will be crucial to validate the functions of each *SPL* gene belonging to different clades to more fully understand the functions of miR156 in determining alfalfa traits.

## Methods

### Plant materials and growth conditions

The WT alfalfa clone N4.4.2 [56] was obtained from Dr. Daniel Brown (Agriculture and Agri-Food Canada). The miR156OE genotypes (A11a and A17) were obtained from our previous study [4]. All the alfalfa plants were grown under greenhouse conditions of 21–23 °C, 16 h light per day, light intensity of 380–450 W/m^2^ (approximately 500 W/m^2^ at high noon time), and a relative humidity of 70 %.

### Propagation of alfalfa by stem cuttings

In order to ensure all plant materials were at a similar developmental stage, prior to vegetative propagation by rooted stem cuttings, alfalfa plants were cut back 2–3 times and grown for 2 months to synchronize growth. For each time point, at least four rooted cuttings (biological replicates) were used in this study. For each replicate, about 3–4 stem sections containing 2 nodes each were cut and inserted into moistened growing media (Pro-Mix, Mycorrhizae^TM^, Premier Horticulture Inc., Woodstock, ON, Canada) in a 5-inche pot. Propagation domes (Ontario grower’s supply, London, ON, Canada) were used to cover the pots which were kept in the greenhouse for 3 weeks to allow rooting from the cut-stem.

### Sequence verification of alfalfa *miR172* precursor

A precursor of *miR172* (approximately 300 bp) was amplified from an alfalfa cDNA template using a pair of primers (Additional file 9: Table S4), which were designed based on a *miR172* precursor in a *M. truncatula* sequence database (mtr-MIR172a: MI0005600, with a verified genome location) [57]. The PCR product was then sequenced, and the sequence was compared to that of *M. truncatula*.

### Extraction of total RNA, reverse transcription and quantitative real time PCR

Different tissues of shoots, leaves, stems and roots of alfalfa plants were collected at 10, 20, 30, and 40 days after synchronizing their growth. Total RNAs were extracted using PowerPlantRNA Isolation Kit (MO BIO Laboratory, Mississauga, ON, Canada) or RNeasy Plant Mini Kit (QIAGEN) and 2 μg was used to generate cDNA through reverse transcription using oligo(dT)_15_ and gene-specific reverse primers with SuperScript® III Reverse Transcriptase kit (Invitrogen^TM^). The transcript levels of *miR156* and *miR172* were analyzed by stem loop qRT-PCR [58] and *SPL* genes by normal qRT-PCR using a CFX96 TouchTM Real-Time PCR Detection System (Bio-Rad). The cDNA was diluted with water (1:3) and qRT-PCR was carried out following PerfeCta SYBR Green FastMix (Quanta Biosciences, Canada) instructions. Each reaction consisted of 2 μl of cDNA template, 0.3 μl each of both gene-specific forward and reverse primers (10 μM) (Additional file 9: Table S4), and topped up to 10 μl ddH_2_O. Two reference genes - acetyl CoA carboxylase 1 (ACC1) and acetyl CoA carboxylase 2 (ACC2) [4] were used to normalize the transcript levels in qRT-PCR. Finally, transcript levels of the respective genes were analyzed using a relative quantification 2^-∆Ct^ method [59].

### RNA sequencing

Total RNA was extracted using PowerPlant® RNA Isolation Kit (MO BIO Laboratories, Inc.), RNA concentrations were determined using a NanoDrop 2000C (Thermo Scientific), and the quality of RNA samples was assessed by agarose gel electrophoresis. Four biological replicates were used for each sample. The RNA library was constructed and sequenced on an Illumina Hi-Seq 2500 using paired-end 101 bp reads at the Centre for Applied Genomics (Sick Kids Hospital, Toronto, Canada). Briefly, before library construction, the integrity of RNA samples was confirmed on an Agilent Bioanalyzer 2100 RNA Nano chip (Agilent Technologies) and an RNA library prepared using the Illumina TruSeq mRNA Library Preparation protocol. The poly(A) RNA from 500 ng of total RNA was enriched with oligo dT beads and then fragmented to convert to double stranded cDNA. One ul of each of the final RNA libraries was loaded on a Bioanalyzer 2100 DNA High Sensitivity chip (Agilent Technologies) to check for size, and the RNA libraries were quantified by qRT-PCR using the Kapa Library Quantification Illumina/ABI Prism Kit protocol (KAPA Biosystems). Finally, six libraries were pooled in one lane with equimolar quantities and sequenced on an Illumina HiSeq 2500 platform using a Rapid Run Mode flowcell (Illumina).

### Differential expression analysis

Using *M. truncatula* as reference genome, differential expression was determined using published protocols [60]. Firstly, the QC analyses were carried out for all the raw reads using FastQC program. Since the quality of all the sequenced reads is identical, two representative results for per-base quality are shown in Additional file 10 Document 2. Raw sequence reads were then 5’ trimmed on quality score (*Q* > 30), adapter sequences removed and short reads dropped using custom Perl scripts. All filtered and properly paired reads were then mapped to the *M. truncatula* genome using TopHat (v2.0.10). The fragment alignments generated by TopHat were used as input files for Cufflink (v2.2.1) and further analyzed through the recommended pipeline to detect the differentially expressed genes between miR156OE and WT [60]. Features with false discovery rate < 0.2 (20 % false positive rate) were considered differentially expressed between conditions. The p-value (>0.005) was used for rejecting the null hypothesis that value2 is equal to value1. More detailed methods and parameters for analyzing RNA-Seq data are listed in Additional file 10: Document 2.

### Transcriptome *de novo* assembly

*De novo* assembly of *M. sativa* transcriptome was performed using the Trinity program as previously described [34]. Compared with other *de novo* assemblers, Trinity is able to recover more full-length transcripts across a broad range of expression levels [34]. Briefly, the 1.11 billion clean RNA-Seq reads mentioned above were used as input for *de novo* assembly. BLAST searches (*E* < 10E-5) were conducted against the NCBI Nr (http:// www.ncbi.nlm.nih.gov/), Swissprot (http://www.expasy.ch/sprot/), KEGG (http://www.genome.jp/kegg/) and COG (http://www.ncbi.nlm.nih.gov/COG/) databases. The used parameters for assembling transcriptome were described in Additional file 10: Document 2.

### GO enrichment analysis

*M. truncatula* GO terms were downloaded from GO Analysis Toolkit and Database for Agriculture Community (AGRI go, http://bioinfo.cau.edu.cn/agriGO/download.php). All the genes identified with significant differential expression (*p* < 0.005) and FC > 2 in this study were used as input to carry out GO enrichment analysis. The enriched GO terms were summarized and plotted following the published REVIGO protocol [61]. The ratios of molecular functions, cellular component and biological process were calculated based on the number of GO terms.

### Detection of miR156 cleavage sites in *MsSPL2*, *MsSPL3*, *MsSPL4*, and *MsSPL9* transcripts

The cleavage sites in alfalfa *SPL* genes were detected using a modified 5’ rapid amplification of cDNA end (5’-RACE) as previously reported [62]. The experiment was conducted using FirstChoice® RLM-RACE Kit (Ambion, Burlington, ON, Canada) following the manufacturer’s instructions with slight modifications. Briefly, PCR products with estimated sizes from inner 5’ RLM-RACE PCR were purified using a gel purification kit (Qiagen, Toronto, ON, Canada) and cloned into a pJET1.2/blunt cloning vector (Fermentas, Ottawa, ON, Canada). At least 20 clones for each *SPL* transcript were subjected to sequencing using a pJET1.2/blunt sequencing primer (Fermentas, Ottawa, ON, Canada).

### Phylogenetic tree construction

The phylogenetic tree was constructed based on an alignment of the SBP-box domain and using publicly available sequences of several representative plants, including *M. sativa*, *M. truncatula*, Arabidopsis and *Glycine Max*. Sequences were downloaded from GeneBank and all the used sequences for the phylogenetic tree are shown in Additional file 11: Document 3. Amino acids were aligned by visualization and nucleotides were subjected to ClustalW2 alignment analysis. The tree was obtained by using FigTreeV1.42. A representation of the conserved SBP domain (amino acids and nucleotides) from the genes included in the phylogenetic tree were analyzed using WebLogo [63].

## Additional files

Additional file 1: Table S1. Assembled alfalfa transcriptome. All the *de no*vo assembled alfalfa transcripts that were obtained from Trinity program. (ZIP 51914 kb) Additional file 2: Table S2. Differentially expressed genes with significant fold change detected between WT and two miR156OE genotypes (A11and A17). Differentially expressed genes between WT and the two miR156OE genotypes A11a and A17. (XLSX 1067 kb) Additional file 3: Table S3. Full list of the components included in cellular component, biological process and molecular function. The full list of the GO terms that correspond to the differentially expressed genes in two genotypes of miR156OE alfalfa plants compared to WT. (XLSX 16 kb) Additional file 4: Figure S1. A representation of the conserved SBP domain (nucleotides) from the genes included in the phylogenetic tree using WebLogo. (TIF 485 kb) Additional file 5: Figure S2. Alignment of *miR172* precursor sequences from *M. truncatula* and *M. sativa.* The precursor sequences share 76.82 % similarity and the mature sequences (red box) are identical. (TIF 1353 kb) Additional file 6: Document 1. “GTAC” elements in the promoter (2000 bp) of *MtLEY* and *MtFUL* genes. Two alfalfa genes (*LFY* and *FUL*) promoter sequences that contain “GTAC” motif. (DOCX 14 kb) Additional file 7: Figure S3. Nucleotide sequences alignment between (a) *M. sativa SPL2/3/4* and Arabidopsis *SPL3/4/5*; (b) *MsSPL9* and *AtSPL9*, respectively. (TIF 3681 kb) Additional file 8: Figure S4. Amino acid sequences alignment between (a) *M. sativa SPL2/3/4* and Arabidopsis *SPL3/4/5*; (b) *MsSPL9* and *AtSPL9*, respectively. (TIF 1205 kb) Additional file 9: Table S4. Primers used in this study. All the primers used in this study, including for PCR, qRT-PCR and gene cloning. (XLSX 15 kb) Additional file 10: Document 2. RNA-Seq data analysis parameters. RNA-Seq raw reads QC results and bioinformatics analysis parameters. (DOCX 35 kb) Additional file 11: Document 3.
*SPL* gene sequences used for the phylogenetic tree. All the *SPL* gene sequences that were used to generate the phylogenetic tree. (DOCX 18 kb)

## Abbreviations

*AP1*: *APETALA1*
DEGs: Differentially expressed genes
*FD*: *FLOWERING LOCUS D*
FT: FLOWERING LOCUS T
*FUL*: *FRUITFULL*
GA: Gibberellic acid
GO: Gene ontology
NLS: Nuclear localization signal
qRT-PCR: Quantitative real time PCR
RNA-Seq: RNA sequencing
*SOC1*: *OVEREXPRESSION OF CONSTANS1*
*SPL*: *SQUAMOSA-PROMOTER BINDING PROTEIN-LIKE*
*TCL1*: *TRICHOMELESS 1*

## References

[1] Xuehui L, Charles B (2012). Applied genetics and genomics in alfalfa breeding. Agronomy.

[2] Sanderson MA, Adler PR (2008). Perennial forages as second generation bioenergy crops. Int J Mol Sci.

[3] Nichols NN, Dien BS, Cotta MA (2010). Fermentation of bioenergy crops into ethanol using biological abatement for removal of inhibitors. Bioresour Technol.

[4] Aung B, Gruber MY, Amyot L, Omari K, Bertrand A, Hannoufa A (2015). MicroRNA156 as a promising tool for alfalfa improvement. Plant Biotechnol J.

[5] Preston JC, Hileman LC (2013). Functional evolution in the plant SQUAMOSA-PROMOTER BINDING PROTEIN-LIKE (SPL) gene family. Front Plant Sci.

[6] Manning K, Tor M, Poole M, Hong Y, Thompson AJ, King GJ, Giovannoni JJ, Seymour GB (2006). A naturally occurring epigenetic mutation in a gene encoding an SBP-box transcription factor inhibits tomato fruit ripening. Nat Genet.

[7] Gandikota M, Birkenbihl RP, Hohmann S, Cardon GH, Saedler H, Huijser P (2007). The miRNA156/157 recognition element in the 3’ UTR of the Arabidopsis SBP box gene SPL3 prevents early flowering by translational inhibition in seedlings. Plant J.

[8] Silva GF F e, Silva EM, Azevedo Mda S, Guivin MA, Ramiro DA, Figueiredo CR, Carrer H, Peres LE, Nogueira FT (2014). microRNA156-targeted SPL/SBP box transcription factors regulate tomato ovary and fruit development. Plant J.

[9] Aung B, Gruber MY, Hannoufa A (2015). The microRNA156 system: a tool in plant biotechnology. Biocatalysis Agric. Biotechnol..

[10] Wang H, Wang H (2015). The miR156/SPL module, a regulatory hub and versatile toolbox, gears up crops for enhanced agronomic traits. Mol Plant.

[11] Shikata M, Koyama T, Mitsuda N, Ohme-Takagi M (2009). Arabidopsis SBP-box genes SPL10, SPL11 and SPL2 control morphological change in association with shoot maturation in the reproductive phase. Plant Cell Physiol.

[12] Stief A, Altmann S, Hoffmann K, Pant BD, Scheible WR, Baurle I (2014). Arabidopsis miR156 regulates tolerance to recurring environmental stress through SPL transcription factors. Plant Cell.

[13] Wu G, Poethig RS (2006). Temporal regulation of shoot development in Arabidopsis thaliana by miR156 and its target SPL3. Development.

[14] Schwarz S, Grande AV, Bujdoso N, Saedler H, Huijser P (2008). The microRNA regulated SBP-box genes SPL9 and SPL15 control shoot maturation in Arabidopsis. Plant Mol Biol.

[15] Gou JY, Felippes FF, Liu CJ, Weigel D, Wang JW (2011). Negative regulation of anthocyanin biosynthesis in Arabidopsis by a miR156-targeted SPL transcription factor. Plant Cell.

[16] Cui LG, Shan JX, Shi M, Gao JP, Lin HX (2014). The miR156-SPL9-DFR pathway coordinates the relationship between development and abiotic stress tolerance in plants. Plant J.

[17] Wei S, Gruber MY, Yu B, Gao MJ, Khachatourians GG, Hegedus DD, Parkin IA, Hannoufa A (2012). Arabidopsis mutant sk156 reveals complex regulation of SPL15 in a miR156-controlled gene network. BMC Plant Biol.

[18] Jiao Y, Wang Y, Xue D, Wang J, Yan M, Liu G, Dong G, Zeng D, Lu Z, Zhu X (2010). Regulation of OsSPL14 by OsmiR156 defines ideal plant architecture in rice. Nat Genet.

[19] Miura K, Ikeda M, Matsubara A, Song XJ, Ito M, Asano K, Matsuoka M, Kitano H, Ashikari M (2010). OsSPL14 promotes panicle branching and higher grain productivity in rice. Nat Genet.

[20] Wang S, Wu K, Yuan Q, Liu X, Liu Z, Lin X, Zeng R, Zhu H, Dong G, Qian Q (2012). Control of grain size, shape and quality by OsSPL16 in rice. Nat Genet.

[21] Xie K, Wu C, Xiong L (2006). Genomic organization, differential expression, and interaction of SQUAMOSA promoter-binding-like transcription factors and microRNA156 in rice. Plant Physiol.

[22] Chuck G, Cigan AM, Saeteurn K, Hake S (2007). The heterochronic maize mutant Corngrass1 results from overexpression of a tandem microRNA. Nat Genet.

[23] Bhogale S, Mahajan AS, Natarajan B, Rajabhoj M, Thulasiram HV, Banerjee AK (2014). MicroRNA156: a potential graft-transmissible microRNA that modulates plant architecture and tuberization in Solanum tuberosum ssp. andigena. Plant Physiol.

[24] Wang Y, Wang Z, Amyot L, Tian L, Xu Z, Gruber MY, Hannoufa A (2015). Ectopic expression of miR156 represses nodulation and causes morphological and developmental changes in Lotus japonicus. Mol Genet Genomics.

[25] Fu C, Sunkar R, Zhou C, Shen H, Zhang JY, Matts J, Wolf J, Mann DG, Stewart CN, Tang Y (2012). Overexpression of miR156 in switchgrass (Panicum virgatum L.) results in various morphological alterations and leads to improved biomass production. Plant Biotechnol J.

[26] Zhou Y, Zhou H, Lin-Wang K, Vimolmangkang S, Espley RV, Wang L, Allan AC, Han Y (2014). Transcriptome analysis and transient transformation suggest an ancient duplicated MYB transcription factor as a candidate gene for leaf red coloration in peach. BMC Plant Biol.

[27] Wei C, Tao X, Li M, He B, Yan L, Tan X, Zhang Y (2015). De novo transcriptome assembly of Ipomoea nil using Illumina sequencing for gene discovery and SSR marker identification. Mol Genet Genomics.

[28] Wang Y, Hua W, Wang J, Hannoufa A, Xu Z, Wang Z (2013). Deep sequencing of Lotus corniculatus L. reveals key enzymes and potential transcription factors related to the flavonoid biosynthesis pathway. Mol Genet Genomics.

[29] Chao J, Chen Y, Wu S, Tian WM (2015). Comparative transcriptome analysis of latex from rubber tree clone CATAS8-79 and PR107 reveals new cues for the regulation of latex regeneration and duration of latex flow. BMC Plant Biol.

[30] Postnikova OA, Hult M, Shao J, Skantar A, Nemchinov LG (2015). Transcriptome analysis of resistant and susceptible alfalfa cultivars infected with root-knot nematode Meloidogyne incognita. PLoS One.

[31] Zhang S, Shi Y, Cheng N, Du H, Fan W, Wang C (2015). De novo characterization of fall dormant and nondormant alfalfa (Medicago sativa L.) leaf transcriptome and identification of candidate genes related to fall dormancy. PLoS One.

[32] O’Rourke JA, Fu F, Bucciarelli B, Yang SS, Samac DA, Lamb JF, Monteros MJ, Graham MA, Gronwald JW, Krom N (2015). The Medicago sativa gene index 1.2: a web-accessible gene expression atlas for investigating expression differences between Medicago sativa subspecies. BMC Genomics.

[33] Xie K, Shen J, Hou X, Yao J, Li X, Xiao J, Xiong L (2012). Gradual increase of miR156 regulates temporal expression changes of numerous genes during leaf development in rice. Plant Physiol.

[34] Grabherr MG, Haas BJ, Yassour M, Levin JZ, Thompson DA, Amit I, Adiconis X, Fan L, Raychowdhury R, Zeng Q (2011). Full-length transcriptome assembly from RNA-Seq data without a reference genome. Nat Biotechnol.

[35] Liang X, Nazarenus TJ, Stone JM (2008). Identification of a consensus DNA-binding site for the Arabidopsis thaliana SBP domain transcription factor, AtSPL14, and binding kinetics by surface plasmon resonance. Biochemistry.

[36] Jung JH, Seo PJ, Kang SK, Park CM (2011). miR172 signals are incorporated into the miR156 signaling pathway at the SPL3/4/5 genes in Arabidopsis developmental transitions. Plant Mol Biol.

[37] Wu G, Park MY, Conway SR, Wang JW, Weigel D, Poethig RS (2009). The sequential action of miR156 and miR172 regulates developmental timing in Arabidopsis. Cell.

[38] Aukerman MJ, Sakai H (2003). Regulation of flowering time and floral organ identity by a MicroRNA and its APETALA2-like target genes. Plant Cell.

[39] Schwab R, Palatnik JF, Riester M, Schommer C, Schmid M, Weigel D (2005). Specific effects of microRNAs on the plant transcriptome. Dev Cell.

[40] Park JH, Shin C (2014). MicroRNA-directed cleavage of targets: mechanism and experimental approaches. BMB Rep.

[41] Li YF, Zheng Y, Addo-Quaye C, Zhang L, Saini A, Jagadeeswaran G, Axtell MJ, Zhang W, Sunkar R (2010). Transcriptome-wide identification of microRNA targets in rice. Plant J.

[42] Usami T, Horiguchi G, Yano S, Tsukaya H (2009). The more and smaller cells mutants of Arabidopsis thaliana identify novel roles for SQUAMOSA PROMOTER BINDING PROTEIN-LIKE genes in the control of heteroblasty. Development.

[43] Cardon GH, Hohmann S, Nettesheim K, Saedler H, Huijser P (1997). Functional analysis of the Arabidopsis thaliana SBP-box gene SPL3: a novel gene involved in the floral transition. Plant J.

[44] Wang JW, Czech B, Weigel D (2009). miR156-regulated SPL transcription factors define an endogenous flowering pathway in Arabidopsis thaliana. Cell.

[45] Yamaguchi A, Wu MF, Yang L, Wu G, Poethig RS, Wagner D (2009). The microRNA-regulated SBP-Box transcription factor SPL3 is a direct upstream activator of LEAFY, FRUITFULL, and APETALA1. Dev Cell.

[46] Jung JH, Ju Y, Seo PJ, Lee JH, Park CM (2012). The SOC1-SPL module integrates photoperiod and gibberellic acid signals to control flowering time in Arabidopsis. Plant J.

[47] Porri A, Torti S, Romera-Branchat M, Coupland G (2012). Spatially distinct regulatory roles for gibberellins in the promotion of flowering of Arabidopsis under long photoperiods. Development.

[48] Yu S, Galvao VC, Zhang YC, Horrer D, Zhang TQ, Hao YH, Feng YQ, Wang S, Schmid M, Wang JW (2012). Gibberellin regulates the Arabidopsis floral transition through miR156-targeted SQUAMOSA promoter binding-like transcription factors. Plant Cell.

[49] Corbesier L, Coupland G (2006). The quest for florigen: a review of recent progress. J Exp Bot.

[50] Corbesier L, Vincent C, Jang S, Fornara F, Fan Q, Searle I, Giakountis A, Farrona S, Gissot L, Turnbull C (2007). FT protein movement contributes to long-distance signaling in floral induction of Arabidopsis. Science.

[51] Preston JC, Hileman LC (2010). SQUAMOSA-PROMOTER BINDING PROTEIN 1 initiates flowering in Antirrhinum majus through the activation of meristem identity genes. Plant J.

[52] Li S, Yang X, Wu F, He Y (2012). HYL1 controls the miR156-mediated juvenile phase of vegetative growth. J Exp Bot.

[53] Yu N, Cai WJ, Wang S, Shan CM, Wang LJ, Chen XY (2010). Temporal control of trichome distribution by microRNA156-targeted SPL genes in Arabidopsis thaliana. Plant Cell.

[54] Yu ZX, Wang LJ, Zhao B, Shan CM, Zhang YH, Chen DF, Chen XY (2015). Progressive regulation of sesquiterpene biosynthesis in Arabidopsis and Patchouli (Pogostemon cablin) by the miR156-targeted SPL transcription factors. Mol Plant.

[55] Yu N, Niu QW, Ng KH, Chua NH (2015). The role of miR156/SPLs modules in Arabidopsis lateral root development. Plant J.

[56] Badhan A, Jin L, Wang Y, Han S, Kowalczys K, Brown DC, Ayala CJ, Latoszek-Green M, Miki B, Tsang A (2014). Expression of a fungal ferulic acid esterase in alfalfa modifies cell wall digestibility. Biotechnol Biofuels.

[57] Krishnakumar V, Kim M, Rosen BD, Karamycheva S, Bidwell SL, Tang H, Town CD (2015). MTGD: The Medicago truncatula genome database. Plant Cell Physiol.

[58] Varkonyi-Gasic E, Wu R, Wood M, Walton EF, Hellens RP (2007). Protocol: a highly sensitive RT-PCR method for detection and quantification of microRNAs. Plant Methods.

[59] Livak KJ, Schmittgen TD (2001). Analysis of relative gene expression data using real-time quantitative PCR and the 2(−Delta Delta C(T)) Method. Methods.

[60] Trapnell C, Roberts A, Goff L, Pertea G, Kim D, Kelley DR, Pimentel H, Salzberg SL, Rinn JL, Pachter L (2012). Differential gene and transcript expression analysis of RNA-seq experiments with TopHat and cufflinks. Nat Protoc.

[61] Supek F, Bosnjak M, Skunca N, Smuc T (2011). REVIGO summarizes and visualizes long lists of gene ontology terms. PLoS One.

[62] Llave C, Xie Z, Kasschau KD, Carrington JC (2002). Cleavage of Scarecrow-like mRNA targets directed by a class of Arabidopsis miRNA. Science.

[63] Crooks GE, Hon G, Chandonia JM, Brenner SE (2004). WebLogo: a sequence logo generator. Genome Res.

