# Supplementary figures and images for: Comparative transcriptome investigation of global gene expression changes caused by miR156 overexpression in Medicago sativa
Source: BMC Genomics. 2016 Aug 19;17:658. doi: 10.1186/s12864-016-3014-6 (PMC4992203; doi:10.1186/s12864-016-3014-6)

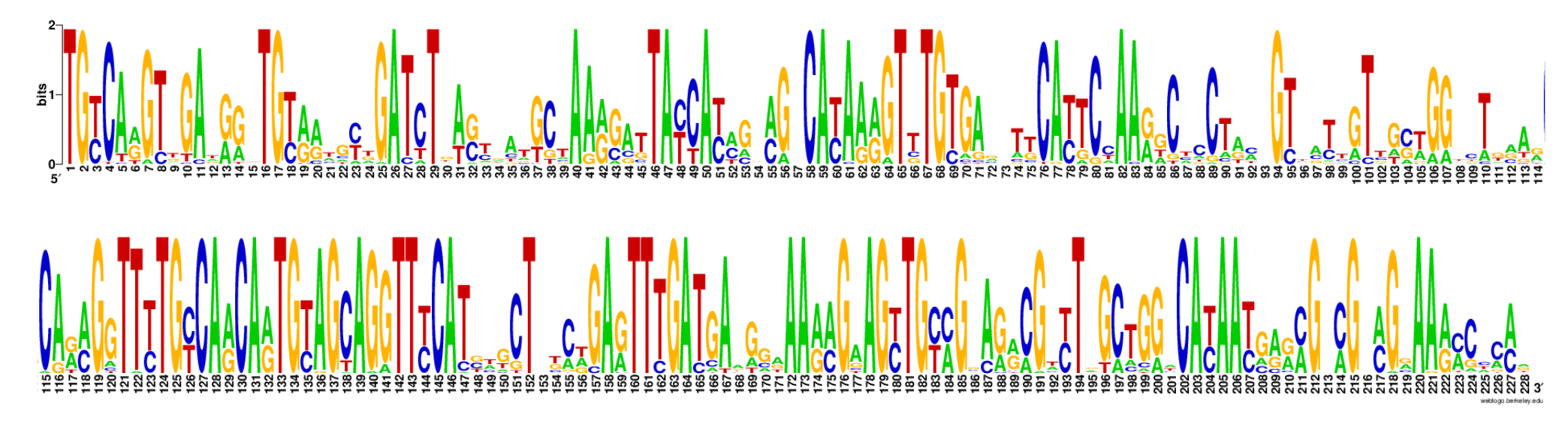

Supplement: Additional file 4: Figure S1. — A representation of the conserved SBP domain (nucleotides) from the genes included in the phylogenetic tree using WebLogo. (TIF 485 kb) [file 12864_2016_3014_MOESM4_ESM.tif]

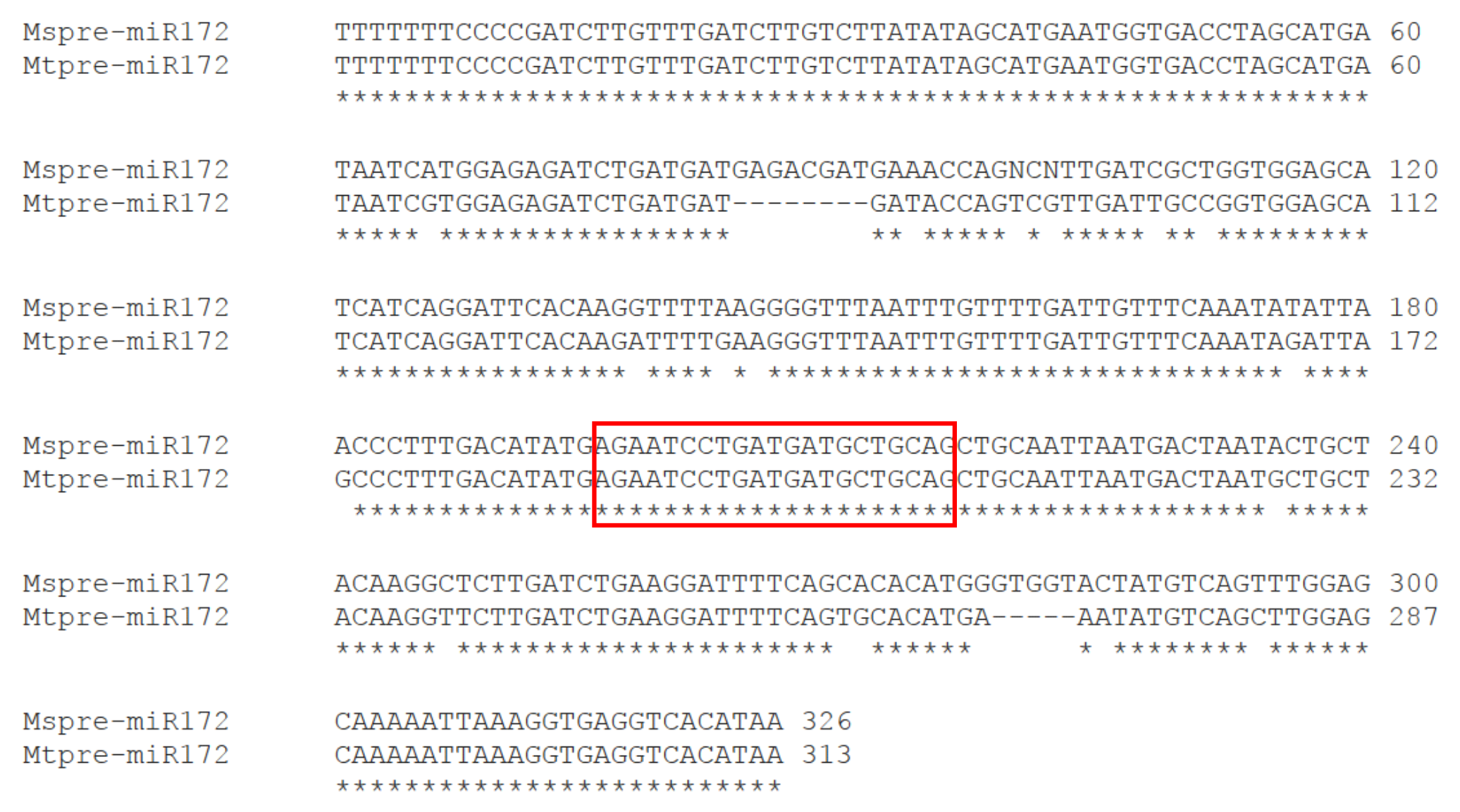

Supplement: Additional file 5: Figure S2. — Alignment of miR172 precursor sequences from M. truncatula and M. sativa. The precursor sequences share 76.82 % similarity and the mature sequences (red box) are identical. (TIF 1353 kb) [file 12864_2016_3014_MOESM5_ESM.tif]

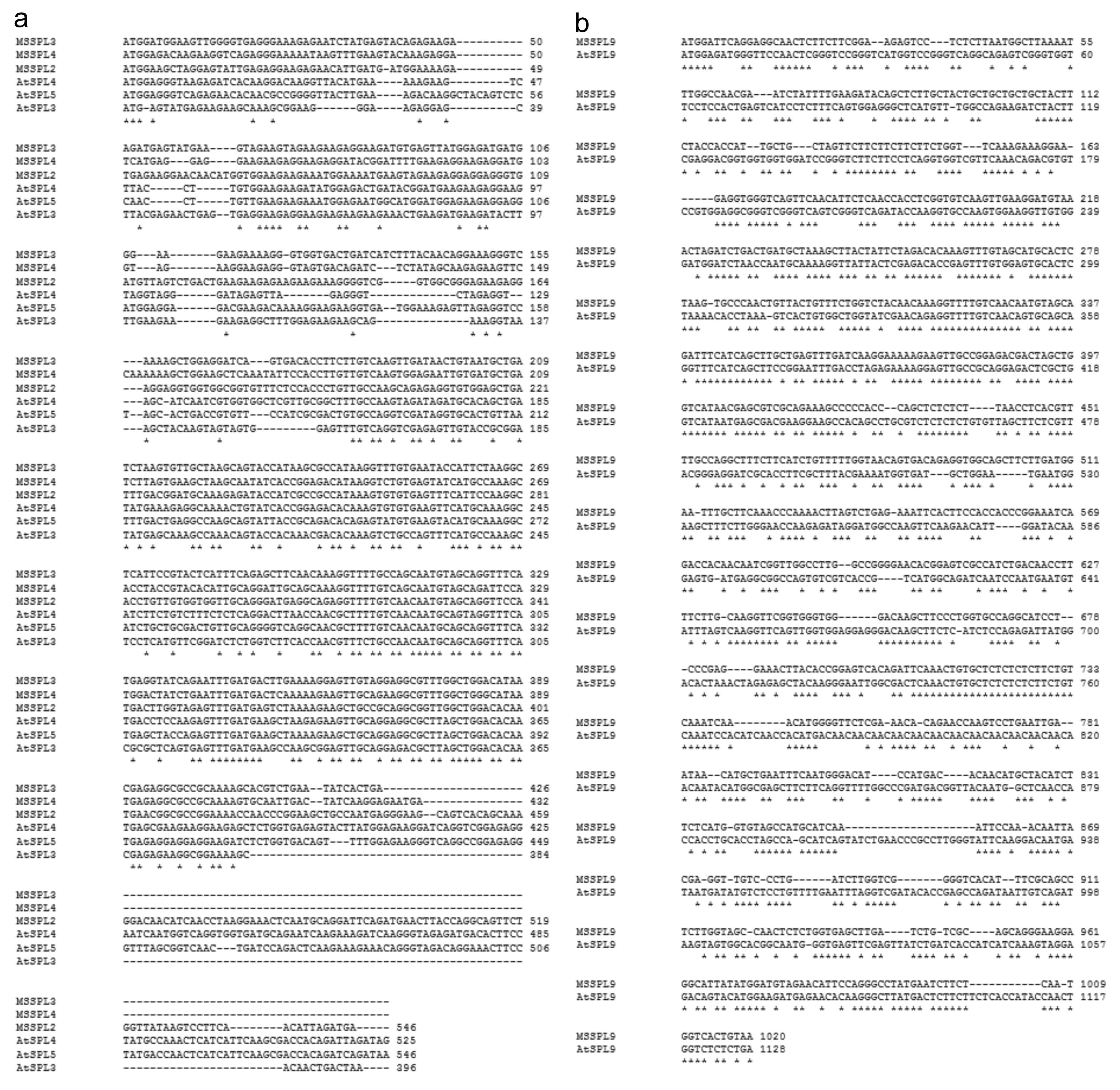

Supplement: Additional file 7: Figure S3. — Nucleotide sequences alignment between (a) M. sativa SPL2/3/4 and Arabidopsis SPL3/4/5; (b) MsSPL9 and AtSPL9, respectively. (TIF 3681 kb) [file 12864_2016_3014_MOESM7_ESM.tif]

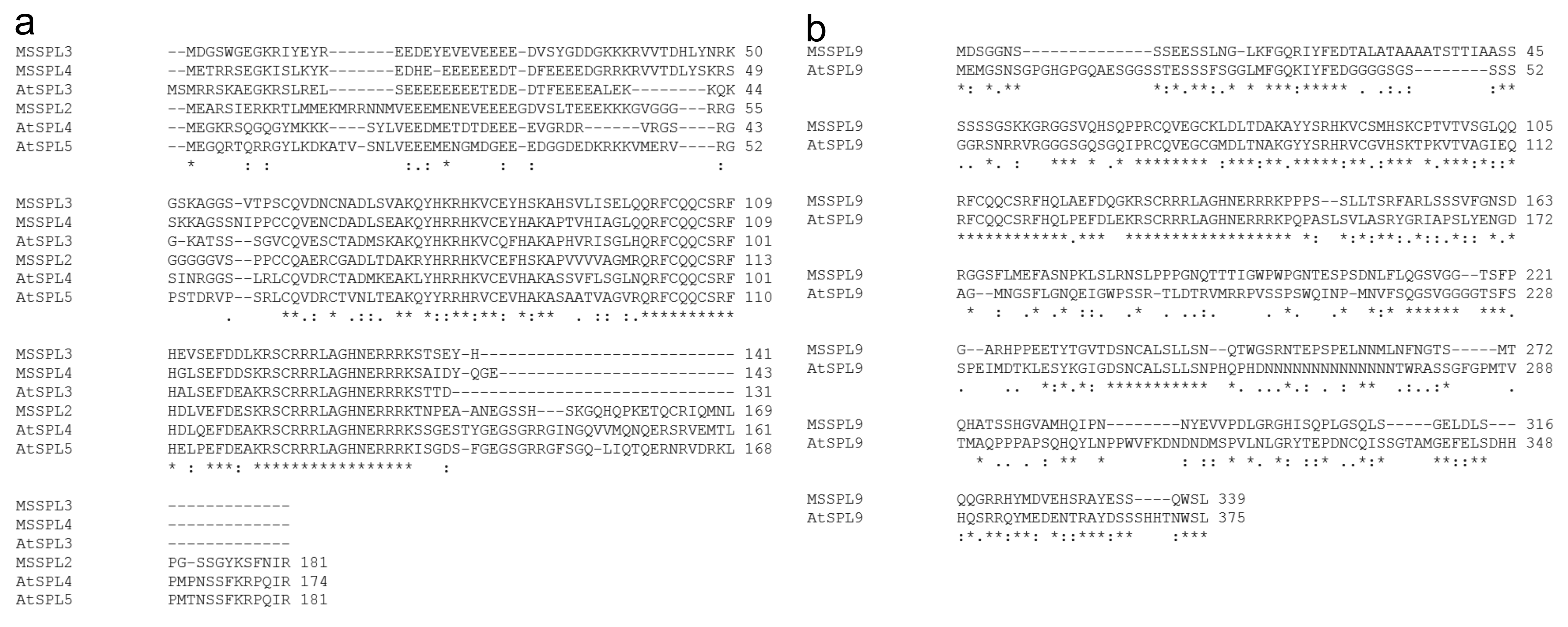

Supplement: Additional file 8: Figure S4. — Amino acid sequences alignment between (a) M. sativa SPL2/3/4 and Arabidopsis SPL3/4/5; (b) MsSPL9 and AtSPL9, respectively. (TIF 1205 kb) [file 12864_2016_3014_MOESM8_ESM.tif]
