# Supplementary material for: Comparative transcriptome investigation of global gene expression changes caused by miR156 overexpression in Medicago sativa
Source: BMC Genomics. 2016 Aug 19;17:658. doi: 10.1186/s12864-016-3014-6 (PMC4992203; doi:10.1186/s12864-016-3014-6)
Supplement: Additional file 6: Document 1. — “GTAC” elements in the promoter (2000 bp) of MtLEY and MtFUL genes. Two alfalfa genes (LFY and FUL) promoter sequences that contain “GTAC” motif. (DOCX 14 kb) [file 12864_2016_3014_MOESM6_ESM.docx]

**Additional file 6: Document 1. “GTAC” elements in the promoter (2000 bp) of *MtLEY* and *MtFUL* genes**

**Medtr3g098560: *LEAFY (LFY)***

TTGACACATATATGATCATTGGTGACAACTCTCTTTGATACTTTATTCTCTCTCTTCATCTCTCTCTCTCTCTCTCTCTCTATATATATATATATATATTGTTATTGTCCAATTAGAAGGGAGGAAAAATGTTGTTACCCAAAATCATTTAAAATTGATTGTCAAAATATCATTCATCTTTAATCAATAAGTCTTCCATTGAGGATCAAGTAGTTTTTGACACTCGCTCTTCTATGCCTACTTCCTCAAATCATTGAGATACGTATAATTGGATTGAATCAAACCAAGTATAATGTTGGATGAGATTGTTCTTCTAAACCTAATCTAAACTACACTATAAACACTCCATTGTATCTGACATTGACACATATAATTCCATTGAATTATAAAAAAAAATCAAGTTATTAACAATATCGACGTGTCATTGTTTGTATCTTGTGCTTCATATATGGTAACCAGCGAGGTGACACCATGCACCCTCCACCTCACTTCACCTCTATTAATGGTTTATGGTAGCTCATGTGTTGGCATGGGTTGGAAATTGGAAGCAATGGTCTTCGAAAGTACACACACATCATGAATTAATCAGCCTTTTTTTTTCAACTTTACAGTGTTTGCTTTAAGACCAACTGGAACAACCTGCAACCCTTGCAGCTGTACGTACTTTGATCCCTGCATCACTGCTTCTGCTTCACATCTCGTCACTCATGAAATAGTAAATAGTAACCCCATTCTTTACTATCATTGTTTCCCCATTAATGTTTTTACTGTTTTTACGTCCTTTTTTTGTCTGTTTCCTTAACGAGCTAGTCTCTCTTCTGTATTACAAACAGCTAAAAACCTCAGTGAGATTTTCTCTGATTCCGTAACTCACCACTACAGTATCAGACACAAGAATCACTCGTGAATCCTCTGCCATCTACCTTTCTTCATTGTAATTCTCACCAAAAATAATTAATAATCAAATTTAATAATTTACCTTTAATTGTTTGGATTGATCACTAGTTACATTAAATCATAATAATAGGCAATAAGGAAAAGTAGCTTGTTGTTTTTCGGGTTACAAACTTACATGCATGCCTTTCACTGACCAGGACCTTACCAACTTTTCCTTATTATTTGCCGCCCTACCCTAAATATAATCATAATGATTGAACATTTGAACTAAATAACCCTAAAAAACCCTAGTATTCTTCATTTAATTAATTTGTTGTTGTTGTTGTTTAAGTAGGCTAATATATATAGGCTTTTACGACAACCCAAAGCATCAATTCATACTTTGTACAACACACAAGCATGCATATGACACATAAGAGCAAGCACAAAAAAAAAAATAACAGTCAAGTCAAATCAGAGGTCAAACCATATACACACAACACAACACAACTGACCCCATCTGGTCGGATATTATTATGGGTAACCACGTCTATCTATAGCCAGTTAAATTAGTTCGGTTTGTTTTGAGGCATTCCAGTTTCAAATCAAATACAGTTCTTAGTCTCACACACAACAAAAGGAAGTCTCCAACAAGCCCTAACCAGTCTTGGGAATAACACTATTTCCTTATAGTTTTGGAAAAACCATGTCAAACAAAGGGTAGTTTTGGAAAATCACGTTATCTTTCTTTCTCCACAGTAAATTTAACCACACCCTCACCACCTTTACGTTTTCTATATCTCCACCTAAACAGTACCATAAATCTTCCACTTCTCATAATTTTCCAAAACCTAACAAACATACACAAAGAAGTTAAAACAGTTTCATTGCTTACCA

**Medtr4g109830: *FRUITFULL (FUL)***

CGTGAAAGCCAAAGTCATCGAGACAGTCTTTGAGAAAGTTCTGATTGACGTTGTCGAAGGTTTTCTCTAGATTAAGTTTGAAAGCAACATCCCTTTCTTCTTCTTGGACTTCCTTGTGGCATGAACAATTAATGGAAAAAATAATATCATTATCAAAAGTATTCGTATATATCAAGCATAAAACTTCTTTGGTAGGGTCAATAATGTTGTTAATGAGGGGGTTGAGGTGGTTAACAAGTACTTTTGTGATAATTTTGTAAATGGTGTTGAAAGGTTAATGAATCTTGTAAGTTCTTTAAAGATGATCAAGATGTCAACTAATTTGAGAATGAAAGTAGTGACAGTTTCAAAGATCGCTATCTTTGAAAATGTCATTTTCTACTATGTATGAAAATTGCTTAAAAACAAAAATGGTACGTATTTAAACCTAAAAAATTAGTAAAAATGTTATTCTATTTTTTATCATTTGTGCATCAAATATATGATAGAATAAGTGTTATCATTATGTCACTAACACACTGATTAGTGTCGAAATGTTTTAAACATTTAAAACTTTATTTTTTAAGGGTCTTGCTAACCATTGCATTCAGGGCAATAGTTAAGGAAGCCAAAAGTAGCATGTTTGCATTAGTTTCAACAACATTTTGACTTTTAAAAAGTTAAATTTATCACTTTTCATCATTTTTCAATGCTATGTTTCTATTTTTATCCCCTTATCCAATACCCCAAGAACAATGGTTAGCATTTTTTTTTTAAAGATAAACATTTAAAACTAGTAAATACCAACATTATAATGGTGAAAAACATTATTAACCTTTGTTTTAAAAGAACTATTACCAACGCATCCTCGTTAACAACACGTTCCAAATTGGCCTAAATAATTACCACATGTTCTCCACCATATTTATCTAGGTACATAGAGTTATTTAAGAGACTGTCAGCTATTATATATATGCACTTACGTATCAAATTGACCACATGGCCAAAAGACCATGTTATCTCTGTTTTTTTTTATATTCTTTTTAAGTAGTTAAGTTTTTCAAAATTCAACTCTTAATTAAAATGAAAAAAATAAAATAAAATTGGAGTTCAAACTCTATCCTCTTAATATCACAATGTCTCTACCAACTATGTTGTACTCTTTTCAAAATTATTTTTCAATTATTAAGAAAGAGAAGTTGTTGATCTTTTTATTCTCCAAATTTATTAGGATGTTTTTATGAAATGAAAAATTAATCAATGTATCTTAAATTTTGAAAATTATCCCATAAAAAGGGACAAGAAAAATCTTGCAAAAGATTTTTTAAATAGGGACAGTTGTAGCATACAATTAAAAATTTCTAAGGGTTATACAGTACAATATTCTTGATTCATTTTAACATATAAAGTAAAAAAAAAATGATAATAATAATAATAAATATGAAACAGAAAATAAAATATTTGATTTCAAGTTACTAAAAATAGTAAGACCACATTTGGACTAGATTCATACTAGAGTCCTCACCTTTTAATTTAAAATAATAAATTATTGATAAATATAGTGACTACTACGTTACAAACTTTATAATTAGAAAAAGTAGTTGAAGAAACTTTTTTGTTTGGTAAAAACATTGAACAGACTTTTAATGAACAAAGTTACCGGATGTTTTATCTTTCCATGACACAAATAAATAAATAAAAAGAATCAAACGAATTGGTACGAGAAACAGTGGGGCCTAAACAAAAAGGACCAAGCCTCCAGCCAATGGTAACGAGACAACGCTTTTGACGAAACCACCATGGTTTCACCAAAACCACCATCGAAATCCTATCAGTGGTTTTATATAATTTGTCTTTTTTTGCTCTTTTTCTCCAGAATATGTTATAATACTGACCACTGATATTTTCCTTTTTTATATAAAACGAAAAAAGAACCAAGGAGCTGCGGTGTATGTGCTCAAAGCCCTTCATTATTTCAAATTAACATTCATTCATTTTCATATCATCATAAATAATAACAA
