# Supplementary material for: Comparative transcriptome investigation of global gene expression changes caused by miR156 overexpression in Medicago sativa
Source: BMC Genomics. 2016 Aug 19;17:658. doi: 10.1186/s12864-016-3014-6 (PMC4992203; doi:10.1186/s12864-016-3014-6)
Supplement: Additional file 10: Document 2. — RNA-Seq data analysis parameters. RNA-Seq raw reads QC results and bioinformatics analysis parameters. (DOCX 35 kb) [file 12864_2016_3014_MOESM10_ESM.docx]

**Additional file 10: Document 2: RNA-Seq data analysis parameters**

**RNA-Seq raw reads QC results (per-base quality)**


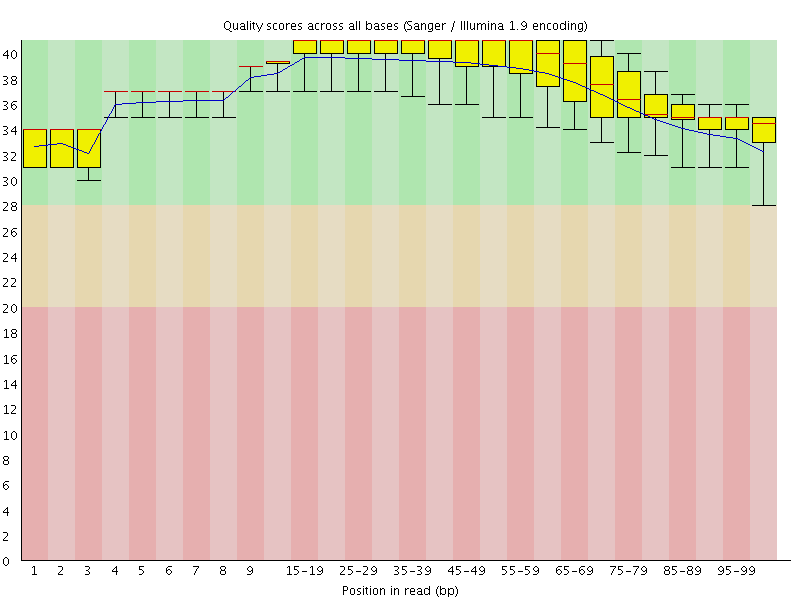

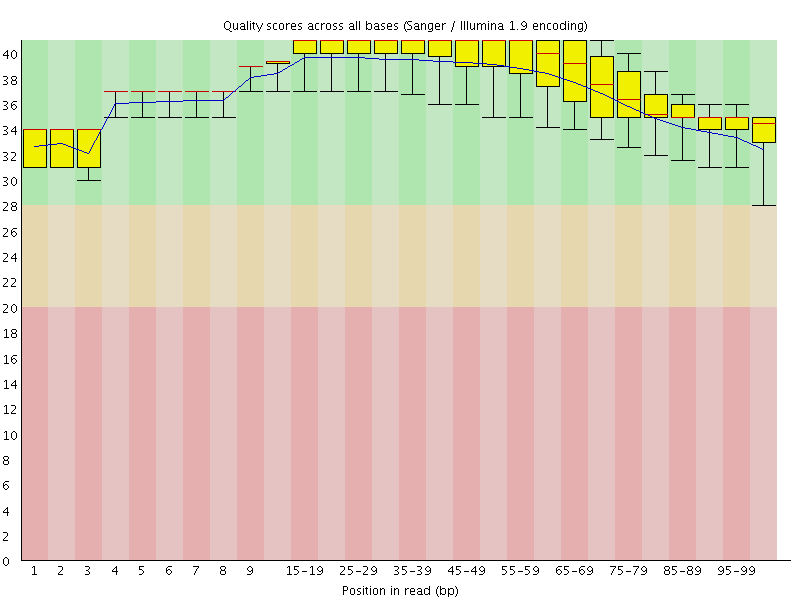


**Methods and parameters for analysing RNA-Seq data using tophat and cufflinks:**

1. Map the reads for each sample to the reference genome

tophat -p 8 -G all.gff3 -r 60 -o 1th R1_001.fastq R2_001.fastq

2. Assemble transcripts for each sample with reference gene structure

cufflinks -g ../all.gff3 -p 8 -o WT_cl WT_tophat/accepted_hits.bam

cufflinks -g ../all.gff3 -p 8 -o miR156OE_cl miR156OE_tophat/accepted_hits.bam

3. Run Cuffcompare on all assemblies to create a single merged transcriptome annotation:

cuffcompare -o OE -s Mt.fa -r ../all.gff3 -R -C WT_cl/transcripts.gtf miR156OE_cl/transcripts.gtf

4. Run Cuffdiff by using the merged transcriptome assembly along with the BAM from Tophat for each replicate

cuffdiff -o diff1 -b Mt.fa -p 8 –L WT,miR156OE -u all_cuffcompare/\all.combined.gtf \./mock_tophat/accepted_hits.bam \./miR156OE_tophat/accepted_hits.bam

**Parameters used for assembling *M.sativa* transcriptome:**

./Trinity --seqType fq --left ../all_F.q30.2.fastq --right ../all_R.q30.2.fastq --output vlMs_90_trinity --CPU 8 --bflyHeapSpaceMax 40G --bflyCPU 8 --min_kmer_cov 5 --max_memory 500G --inchworm_cpu 8 --min_glue 10 --min_per_id_same_path 90 > vlMs_90_trinity.log &)
