# Supplementary material for: Comparative transcriptome investigation of global gene expression changes caused by miR156 overexpression in Medicago sativa
Source: BMC Genomics. 2016 Aug 19;17:658. doi: 10.1186/s12864-016-3014-6 (PMC4992203; doi:10.1186/s12864-016-3014-6)
Supplement: Additional file 11: Document 3. — SPL gene sequences used for the phylogenetic tree. All the SPL gene sequences that were used to generate the phylogenetic tree. (DOCX 18 kb) [file 12864_2016_3014_MOESM11_ESM.docx]

**Additional file 11: Document 3: SPL gene sequences used for the phylogenetic tree**

>AtSPL1

tgtcaggtcgaaaactgtgaagctgatcttagtaaagttaaggattatcatagacgccataaggtctgtgagatgcattc

caaggctactagtgccactgtcggaggtatcttgcagcgcttttgtcagcaatgtagtaggttccatcttcttcaggagt

ttgatgaaggaaagagaagttgtcgtagacgtttggctggccataataaacgtcggaggaaaacaaat

>AtSPL2

tgtcaagttgaaggctgtaatcttgatctttcatcagctaaagactatcatcggaaacataggatttgtgaaaatcattc

aaagtttcctaaagtcgttgtgagtggcgtagagcgtcggttctgccaacaatgtagcaggttccactgtctctctgagt

ttgatgagaagaaacgtagctgtcgccgacgtctctcagatcacaatgcaagacgtcgcaagccaaat

>AtSPL3

tgtcaggtcgagagttgtaccgcggatatgagcaaagccaaacagtaccacaaacgacacaaagtctgccagtttcatgc

caaagctcctcatgttcggatctctggtcttcaccaacgtttctgccaacaatgcagcaggtttcacgcgctcagtgagt

ttgatgaagccaagcggagttgcaggagacgcttagctggacacaacgagagaaggcggaaaagcaca

>AtSPL4

tgccaagtagatagatgcacagctgatatgaaagaggcaaaactgtatcaccggagacacaaagtgtgtgaagttcatgc

aaaggcatcttctgtctttctctcaggacttaaccaacgcttttgtcaacaatgcagtaggtttcatgacctccaagagt

ttgatgaagctaagagaagttgcaggaggcgcttagctggacacaatgagcgaagaaggaagagctct

>AtSPL5

tgccaggtcgataggtgcactgttaatttgactgaggccaagcagtattaccgcagacacagagtatgtgaagtacatgc

aaaggcatctgctgcgactgttgcaggggtcaggcaacgcttttgtcaacaatgcagcaggtttcatgagctaccagagt

ttgatgaagctaaaagaagctgcaggaggcgcttagctggacacaatgagaggaggaggaagatctct

>AtSPL6

tgtcaagtttatgggtgtagtaaggatctgagctcttcgaaagattaccacaaaaggcatagagtttgcgaggctcattc

gaaaacttctgtggtcatagttaatggtcttgaacagaggttttgtcaacagtgcagcaggtttcatttcctctcagagt

ttgatgatggcaaaagaagttgcagaaggcgattagccggtcacaatgaacgaagaaggaaacctgca

>AtSPL7

tgtcaggttccggattgtgaagcggatattagcgagctcaaagggtaccataagaggcatagggtttgtctccgttgcgc

taccgccagctttgttgtgcttgatggagagaataagagatactgtcaacagtgtggaaagtttcatttgctcccggact

ttgatgaaggaaaacgcagctgtcggagaaagctagagcgtcacaacaacagacggaaaaggaaacct

>AtSPL8

tgccaagcagagggatgcaacgcggatctgagccacgcgaaacactaccacagaaggcacaaagtgtgcgaattccactc

aaaagcatcgacggttgtagccgccggactaagccaaaggttttgccagcaatgcagcaggttccatttgctgtcggaat

tcgacaacgggaaacggagctgccgtaagcgacttgctgaccataaccgccgccgccgtaaatgtcac

>AtSPL9

tgccaagtggaaggttgtgggatggatctaaccaatgcaaaaggttattactcgagacaccgagtttgtggagtgcactc

taaaacacctaaagtcactgtggctggtatcgaacagaggttttgtcaacagtgcagcaggtttcatcagcttccggaat

ttgacctagagaaaaggagttgccgcaggagactcgctggtcataatgagcgacgaaggaagccacag

>AtSPL10

tgccaaattgatggctgtgagctggatctctcatcttctaaggattatcatcgcaagcatagagtctgcgaaacgcattc

aaagtgcccaaaagttgttgtgagtggcctggaacgtcgtttctgccaacagtgtagcaggttccatgctgtctcagaat

ttgatgaaaagaaacgaagctgccgcaaacgtctttctcatcataatgcaaggcgtcgcaagccacaa

>AtSPL11

tgccaaattgatggctgtgaactggatctctcatctgctaagggttatcatcgtaagcacaaagtctgcgaaaagcattc

aaagtgcccaaaagttagcgtgagtggcctggaacgtcggttctgccaacagtgtagcaggttccatgctgtctctgaat

ttgatgagaagaaacgaagctgccgaaaacgtctttctcatcataatgcgaggcgtcgtaagccacaa

>AtSPL12

tgtcaggtagacaactgtggagctgatttaagcaaagttaaggattatcatagacgtcataaggtctgtgagattcattc

taaagctactactgcacttgttggaggaattatgcagcggttttgtcagcaatgtagtaggtttcatgtgcttgaagagt

ttgatgagggaaagagaagttgccgtagacgtttggctgggcataataagcgtagaagaaaagcaaat

>AtSPL13

tgtcttgttgatggatgtgattctgattttagtaattgtagagagtatcataagagacataaagtttgtgatgttcattc

aaaaactcctgtggttactattaatggtcataaacagaggttttgtcaacaatgcagcaggtttcatgctttggaggagt

ttgatgaagggaagagaagttgtaggaaacgtcttgatggacataatcgaagacgacggaagccgcag

>AtSPL14

tgtcaggttgataattgtactgaagatttgtctcatgctaaggattatcatagaaggcataaagtttgtgaagttcatag

taaagctactaaagctcttgttggtaaacagatgcagaggttttgccaacagtgtagcaggtttcatctgctttctgagt

ttgatgaggggaagagaagttgtaggcgtagattggctggccataatcgacggaggaggaaaactacg

>AtSPL15

tgccaagtggaaggttgtagaatggatctaagcaatgttaaagcttattactcgagacacaaagtttgttgcattcactc

taaatcatctaaagtcattgtctctggtcttcatcaaaggttttgtcaacaatgtagcaggtttcaccagctttctgagt

ttgacttggagaaaagaagttgtcgcagaagactcgcttgtcataacgaacgacgaagaaaaccacaa

>AtSPL16

tgtcaggttgataattgtaaggaagatttatcaattgctaaggattatcatagaagacataaagtttgtgaggttcatag

caaagctactaaagctcttgttgggaaacagatgcagaggttttgccaacagtgtagcaggtttcatctgctttctgagt

ttgatgaggggaagagaagttgtaggcgtagattggatggtcataacaggcggaggagaaaaacacag

>GmSPL1a

tgtcaagttgatggttgtagcgctgatctaagtgaagctaagccctaccataggcgtcacaaggtttgtgagtaccatgc

caaggctcctgccgtactcattggagaccagcaccaacggttttgccaacaatgtagtaggtttcatgagctatcagaat

tcgatgactcaaaaaggagttgcagaagacgtttggctggacataatgagaggcgtcgcaaaaatgca

>GmSPL1b

tgccaagtggacaattgtgatgctgatctgagtgaagctaagcagtaccacagaagacacaaggtttgtgagtaccatgc

caaggctccttccgtacacatggcagggctgcaacaaaggttttgccaacaatgtagcagattccatgtgctatcagaat

ttgatgactcaaagaggagttgtagaacgcggttggctgggcataatgagaggcgtcgcaaatatgca

>GmSPL2

tgtcaggcagagatatgtggtgctgatttgactgttgcaaagaggtaccatcgccgtcataaggtgtgtgagcttcattc

caaggctccttctgtgatggttgcaggactgaggcagaggttttgccagcaatgtagcaggttccatgagctggcagagt

ttgatgaagctaaaagaagctgccggagacgcttggccagacacaacgagcggcgccggaaaagcaac

>GmSPL6

tgtcaagtttatggttgtaacatggatcttagctcctcaaaagattaccacaaaaggcataaagtttgtgatgctcactc

caaaacagctaaagttattgtaaatggaattgaacagaggttttgtcagcagtgcagcaggtttcatttgctagctgagt

tcgatgatggtaagcgcagttgtcgcaggcgtctagcaggacacaatgaacgccgaaggaaaccccaa

>GmSPL8

tgccaggccgagggttgcaatgctgatctgtcgcaagccaagcactatcaccgccgccacaaggtgtgcgagtttcactc

caaagccgccaccgtcatcgccgccggcttgactcagcgattctgccagcaatgcagcaggttccatcttctttctgagt

ttgataacggaaaacgtagctgcaggaagagattggcagatcataatcgccgcagaagaaaaactcaa

>GmSPL9

tgtcaggttgagggctgcaaagtagatctgagtggtgctaaggcttactattctaggcacaaagtttgtaccatgcactc

caagttccctactgttattgttgctggtttggagcaaaggttttgccaacagtgtagcaggtttcatctgctttctgaat

ttgatgaaggaaaacgaagctgccgcaggcgacttgctggtcataatgagcgccgacgaaagcccccg

>GmSPL9b

tgtcaagttgaagggtgcaaagtagatctgagtgatgcaaaagcttactattctagacacaaggtctgtggcatgcactc

taaatccccttcagtcattgttgctggtcttcaacaaaggttttgtcaacagtgtagcaggtttcatcagcttcctgagt

ttgatcaaggaaaaagaagttgccgtaggcgactagctggccataatgaacgtcggagaaagccccca

>GmSPL12b

tgtcaggcagagatgtgtggtgctgatttgactgttgcaaagaggtaccatcgccgccataaggtgtgtgagcttcattc

caaggctccttctgtgatggttgcaggactgaggcagaggttttgccagcaatgtagcaggttccaagagctggcagagt

ttgatgaagctaaaagaagctgccggagacgcttggccagacataacgagcggcgccggaaaagcaat

>GmSPL13

tgccttgtggatgggtgcaattcagatcttagcaattgtagagattatcataggcgccataaggtgtgtgagctccattc

caagaccccagaggtcacaattggtggcttcaagcaaaggttctgccaacaatgtagcaggttccattcgctggagcaat

ttgatgaaagaaaaagaagctgcagaaaacgtttagatggacacaatagaaggagaagaaagccccag

>GmSPL14

tgccaggtggacaactgcagagaggatctgtcgaaggcgaaggactatcaccggagacacaaggtgtgtgaggctcatag

caaggcctccaaagcgcttctcgcgaatcaaatgcaaagattctgccagcagtgtagcaggtttcatcctctctcggagt

ttgatgaggggaagcggagctgccgccggagactcgccggacacaaccggcgccggcggaagacgcag

>GmSPL16

tgttctgttgatggatgcaattctgacctcagtgattgcagagattatcacaggcgccatagggtctgcgaaaagcactc

caaaaccccggttgtgttggtgggggggaaacaacagaggttctgccaacaatgcagcaggtttcattcacttggggagt

ttgatgaggttaagaggagttgtaggaaacggcttgacgggcataacaggcgccggaggaaacctcag

>GmSPL17

tgtcaggttgcgggctgcaaagtagatctgagtggtgctaaggcttactattctaggcacaaagtttgtgccatgcactc

caagtcccctactgtcactgttgctggtttggagcaaaggttttgccaacagtgtagcaggtttcatctgctttctgaat

ttgatcaaggaaaacgtagctgccgcaagcgacttgctggccataatgagcgccgacgaaagccccca

>MsSPL2

tgccaagcagagaggtgtggagctgatttgacggatgcaaagagataccatcgccgccataaagtgtgtgagtttcattc

caaggcacctgttgtggtggttgcagggatgaggcagaggttttgtcaacaatgtagcaggttccatgacttggtagagt

ttgatgagtctaaaagaagctgccgcaggcggttggctggacacaatgaacggcgccggaaaaccaac

>MsSPL3

TGTCAAGTTGATAACTGTAATGCTGATCTAAGTGTTGCTAAGCAGTACCATAAGCGCCATAAGGTTTGTGAATACCATTC

TAAGGCTCATTCCGTACTCATTTCAGAGCTTCAACAAAGGTTTTGCCAGCAATGTAGCAGGTTTCATGAGGTATCAGAAT

TTGATGACTTGAAAAGGAGTTGTAGGAGGCGTTTGGCTGGACATAACGAGAGGCGCCGCAAAAGCACG

>MsSPL4

TGTCAAGTGGAGAATTGTGATGCTGATCTTAGTGAAGCTAAGCAATATCACCGGAGACATAAGGTCTGTGAGTATCATGC

CAAAGCACCTACCGTACACATTGCAGGATTGCAGCAAAGGTTTTGTCAGCAATGTAGCAGATTCCATGGACTATCTGAAT

TTGATGACTCAAAAAGAAGTTGCAGAAGGCGTTTGGCTGGGCATAATGAGAGGCGCCGCAAAAGTGCA

>MsSPL6

TGCCAAGTTTATGGTTGTAACATGGATCTTAGCTCCTCGAAAGATTACCACAAAAGGCATAAAGTTTGTGACGTTCACTC

CAAGACAGCTAAAGTTATCGTCAATGGTGTTGAACAGAGGTTTTGTCAGCAGTGCAGCAGGTTCCATCTGGTAACTGAGT

TCGACGATGGTAAGCGCAGTTGTCGCAGGCGTCTAGCTGGACACAATGAGCGCCGAAGGAAACCTCAG

>MsSPL9

TGTCAAGTTGAAGGATGTAAACTAGATCTGACTGATGCTAAAGCTTACTATTCTAGACACAAAGTTTGTAGCATGCACTC

TAAGTGCCCAACTGTTACTGTTTCTGGTCTACAACAAAGGTTTTGTCAACAATGTAGCAGATTTCATCAGCTTGCTGAGT

TTGATCAAGGAAAAAGAAGTTGCCGGAGACGACTAGCTGGTCATAACGAGCGTCGCAGAAAGCCCCCA

>MsSPL12

TGCCAGGTGGAAGGTTGTGGCCTCGACCTCTCTTTGGCTAAAGATTACCATCGGAAACATAGAATTTGTGACAGTCATTC

CAAATCGCCTGTGGTGGTAGTAGCTGGTTTGGAGCGTCGATTTTGCCAGCAGTGTAGCAGGTTCCATGATCTCTCAGAGT

TTGATGATAAAAAAAGAAGCTGCAGACGCCGTCTTTCAGATCACAATGCAAGGCGTCGCAAACCTCAG

>MsSPL13

TGTCTTGTGGATGGGTGCAATTCTGATCTTAGTAATTGTAGAGATTATCATAGGCGTCATAAGGTTTGTGAACTTCATTC

TAAGACTCCAGAGGTTACAATTTGTGGCCTTAAACAAAGGTTCTGCCAACAGTGTAGCAGGTTTCATTCGCTGGAGCAAT

TTGATGAAAGAAAAAGAAGCTGTAGGAAACGTTTAGATGGACACAACCGAAGAAGAAGAAAACCACAG

>MtSPL1a

tgtcaggtggaggattgtcgcgcagatcttaacaacgcgaaagattatcaccgacgtcataaagtttgtgagatacattc

aaaggctagtaaagcccttgtcggaaatgcgatgcagcggttctgccaacaatgtagtaggtttcacttgcttcaagagt

ttgatgaaggaaagagaagctgtcgaagacgtttggcaggccataataaaaggaggagaaaaacaaat

>MtSPL1b

tgtcaggttgaagactgtggtgcagatctgagtagaggcaaggattatcacagacgtcataaagtttgtgagatgcattc

taaggctagtagggctcttgtgggaaatgcaatgcagaggttttgtcaacagtgtagtaggtttcacatacttgaagagt

tcgatgaaggaaagagaagctgtcgaagacgcttggctggccataacaaacgtagaaggaaaacaaat

>MtSPL2

tgccaggtggaaggttgtggcctcgacctctcttttgctaaagattaccatcggaaacatagaatttgtgacagtcattc

caaatcgcctgtggtggtagtagctggtttggagcgtcgattttgccagcaatgtagcaggttccatgatctctcagagt

ttgatgataaaaaaagaagctgcagacgccgtctttcagatcacaatgcaaggcgtcgcaaacctcag

>MtSPL6

tgccaagtttatggttgtaatatggatcttagctcctcgaaagattaccacaaaaggcataaagtttgtgatgttcactc

caagacagctaaagttatcgtcaatggtgttgaacagaggttttgtcagcagtgcagcaggttccatctagtagctgagt

tcgacgatggtaagcgcagttgtcgcaggcgtctagctggacacaatgagcgccgaaggaaacctcag

>MtSPL8

tgtcaagccgaaggttgcaatgctgatctatctcaggctaagcattaccaccgccgccacaaagtttgtgagtttcactc

gaaggcagccaccgtcgttgcagctgggttgactcagcggttctgccagcaatgcagcaggttccatcttctatctgagt

ttgataatggaaaacgtagctgcaggaagagattggctgatcataatcgtcgtaggagaaaaactcag

>MtSPL9

tgtcaagttgaaggatgtaaactagatctgactgatgctaaagcttactattctagacacaaagtttgtagcatgcactc

taaatcccctactgttactgtttctggtcttcaacaaaggttttgtcaacaatgtagcagatttcatcagcttgctgagt

ttgatcaaggaaaaagaagttgtcggagacgactagctggtcataatgagcgtcgcagaaagccccca

>MtSPL14

tgtcttgtggatgggtgcaattctgatcttagtaattgtagagattatcataggcgtcataaggtttgtgaacttcattc

caagactccagaggttacaatttgtggccttaaacaaaggttctgccaacagtgtagcaggtttcattcactggagcaat

ttgatgaaagaaaaagaagttgtaggaaacgtttagatggacacaaccgaaggagaagaaaacctcaa
